# Robotic Computer‐Assisted Dental Implant Surgery Versus Free‐Hand and Guided Techniques: A Systematic Review and Meta‐Analysis of Randomized Controlled Trials

**DOI:** 10.1002/cre2.70460

**Published:** 2026-09-19

**Authors:** Sultan Ainoosah, Redhwan Saleh Al‐Gabri, Omar M. Alaryani, Ahmad Abdulkareem Alnazzawi, Ahmed E. Farghal, Ahmed Yaseen Alqutaibi

**Affiliations:** ^1^ Substitutive Dental Science Department, College of Dentistry Taibah University Madinah Saudi Arabia; ^2^ Department of Prosthodontics, Faculty of Dentistry Ibb University Ibb Yemen; ^3^ Department of Prosthodontics, Faculty of Dentistry National University Ibb Yemen

**Keywords:** dynamic navigation, free‐hand surgery, positional trueness, randomized controlled trials, robotic implant surgery, static guided surgery

## Abstract

**Background:**

Robotic computer‐assisted implant surgery (r‐CAIS) aims to improve implant placement trueness, but existing reviews include heterogeneous designs, and no review has exclusively synthesized RCTs to date. This review aimed to evaluate the positional trueness and clinical outcomes of r‐CAIS compared with free‐hand implant surgery (FHIS), dynamic computer‐assisted implant surgery (d‐CAIS), and static computer‐assisted implant surgery (s‐CAIS), through a systematic review and meta‐analysis of RCTs.

**Materials and Methods:**

This systematic review was conducted according to PRISMA 2020 guidelines. Electronic searches were performed in major biomedical databases from inception to March 2026. Only RCTs were eligible. Risk of bias was assessed using the RoB 2 tool and certainty of evidence was rated using the GRADE framework. Meta‐analyses were performed using a random‐effects model where feasible.

**Results:**

Seven RCTs enrolling 529 implants were included. Moderate‐certainty evidence demonstrated significantly improved positional trueness with r‐CAIS over FHIS across all deviation parameters, with the largest effect observed for angular deviation (MD = −4.56°; 95% CI −6.25 to −2.86). High certainty evidence showed a significant angular deviation advantage of r‐CAIS over d‐CAIS (MD = −0.77°; 95% CI: −1.33 to −0.21), while platform and apical deviations did not differ significantly. All comparisons with s‐CAIS were non‐significant, rated low to very low certainty from a single small trial. Operative time evidence was very low certainty, precluding any directional conclusion. Early implant retention ranged from 93.3% to 100% across all modalities with no meaningful between‐group differences.

**Conclusions:**

r‐CAIS improves implant positional trueness compared with free‐hand surgery, supported by moderate certainty evidence, with a high certainty angular deviation advantage over dynamic navigation. However, improved trueness has not been shown to translate into better clinical or patient‐reported outcomes. Wider clinical use should depend on long‐term, multicenter RCTs using standardized patient‐centered outcomes.

**Abbreviations**
CAIS: computer‐assisted implant surgery
CENTRAL: Cochrane Central Register of Controlled Trials
d‐CAIS: dynamic computer‐assisted implant surgery
FHIS: free‐hand implant surgery
GRADE: Grading of Recommendations Assessment, Development and Evaluation
ID‐COSM: Implant Dentistry Core Outcome Set and Measurement
MeSH: Medical Subject Headings
OHIP‐14: Oral Health Impact Profile‐14
PICOS: Population, Intervention, Comparator, Outcomes, Study Design
PRISMA: Preferred Reporting Items for Systematic Reviews and Meta‐Analyses
PROSPERO: International Prospective Register of Systematic Reviews
RCT: randomized controlled trial
RoB 2: risk of bias 2 tool
r‐CAIS: robotic computer‐assisted implant surgery
s‐CAIS: static computer‐assisted implant surgery

## 1 Introduction

Accurate three‐dimensional positioning of dental implants is critical for achieving optimal prosthetic outcomes, preserving peri‐implant tissues, and minimizing mechanical and biological complications. Deviations from planned position may compromise long‐term outcomes (Miadili et al. 2025; Ding et al. 2023; Alqutaibi et al. 2025a). Free‐hand implant surgery (FHIS) remains widely practiced; however, it is inherently operator‐dependent and susceptible to cumulative errors in angulation and depth control (Schnutenhaus et al. 2021).

To address FHIS limitations, computer‐assisted implant surgery (CAIS), including static and dynamic navigation systems, has been introduced to enhance surgical trueness (Alqutaibi et al. 2025a; Chen et al. 2023; Yang et al. 2024; Alqutaibi et al. 2025b; Werny et al. 2025). More recently, robotic computer‐assisted implant surgery (r‐CAIS) has emerged as an advanced technology designed to further reduce operator variability through haptic guidance, optical tracking, and varying levels of autonomy (Qiao et al. 2023; Li et al. 2024; Wang et al. 2024; Ma et al. 2025).

Recent randomized clinical trials (RCTs) have consistently demonstrated that r‐CAIS provides superior placement trueness compared with FHIS and, in some cases, dynamic navigation or static guidance approaches. Studies published between 2024 and 2026 reported improvements in coronal, apical, linear, and angular deviations using semi‐active, semi‐autonomous, autonomous, and collaborative robotic systems, with maintained accuracy even in complex cases involving bone augmentation (Yang et al. 2024; Shi et al. 2024, 2025; Yang et al. 2025; Chen et al. 2025; Wei et al. 2025; Dash et al. 2026).

Despite the growing body of evidence, previously published systematic reviews in this field have often combined heterogeneous study designs, including in vitro experiments, (Jain et al. 2023; Pisla et al. 2025; Yang and Li 2024; Khaohoen et al. 2025; Khan et al. 2024; Wang et al. 2025) animal studies, (Wang et al. 2025) cadaveric models, (Yang and Li 2024) technical feasibility reports, (Khaohoen et al. 2024; Luo et al. 2025) case series, (Khaohoen et al. 2024; Luo et al. 2025) and non‐randomized clinical trials (Jain et al. 2023; Pisla et al. 2025; Yang and Li 2024; Khaohoen et al. 2025; Khan et al. 2024; Wang et al. 2025; Khaohoen et al. 2024; Luo et al. 2025; Wu et al. 2024; Jia et al. 2026).

Importantly, no prior systematic review has exclusively synthesized evidence from RCTs focusing specifically on robotic implant placement. By restricting inclusion to RCTs alone, the present review eliminates the overestimation of effect sizes arising from controlled laboratory conditions and selection bias inherent in non‐randomized designs — a methodological distinction that sets this synthesis apart from previous work and yields a clinically grounded, internally valid estimate of effectiveness.

Accordingly, the objective of this systematic review was to evaluate, using only RCTs, whether r‐CAIS improves positional trueness and clinical outcomes compared with free‐hand, dynamic navigation, and static guided techniques. Accordingly, the null hypothesis of this systematic review was that r‐CAIS does not differ from free‐hand, dynamic navigation, or static guided techniques in terms of implant placement trueness and clinical outcomes when evaluated using RCTs.

## 2 Materials and Methods

### 2.1 Study Design and Protocol Registration

This systematic review and meta‐analysis was conducted and reported in accordance with the Preferred Reporting Items for Systematic Reviews and Meta‐Analyses (PRISMA) 2020 statement, (Page et al. 2021) and the protocol for this review was registered in the International Prospective Register of Systematic Reviews (PROSPERO) database (CRD420261326729). The methodology was established a priori, including eligibility criteria, search strategy, study selection, data extraction, and risk of bias assessment.

### 2.2 Eligibility Criteria

Studies were selected according to predefined inclusion criteria based on the PICOS framework. Eligible studies were RCTs evaluating r‐CAIS in human patients undergoing dental implant placement. The intervention of interest was r‐CAIS, including semi‐autonomous, collaborative, or fully autonomous robotic systems. Comparators included FHIS (defined here as digitally planned implant placement executed without active robotic, navigational, or static guidance constraint), d‐CAIS, and s‐CAIS. Only full‐text articles published in peer‐reviewed journals were considered eligible.

Studies were excluded if they were non‐randomized, in vitro, animal‐based, case reports, reviews, technical notes without clinical outcomes, or did not report quantitative trueness outcomes.

The primary outcome was implant positional trueness, assessed through platform global deviation, apical global deviation, and angular deviation. Secondary outcomes included operative time, postoperative pain (VAS), postoperative edema, intraoperative and postoperative complications, and early implant retention rate. Full outcome definitions are provided in Table 1.

**Table 1:** Outcome measures: definitions and clinical context.

Outcome	Definition and clinical context
**Primary outcomes — Positional Trueness**; trueness is how close the test values are to a known standard.	
Platform (coronal) global deviation	The three‐dimensional Euclidean distance (mm) between the center of the planned implant platform and the center of the actually placed implant platform, as measured by superimposing postoperative imaging onto the preoperative digital plan. Represents the overall linear discrepancy at the coronal (neck) level of the implant, irrespective of direction.
Apical global deviation	The three‐dimensional Euclidean distance (mm) between the planned and actual position of the implant apex (tip). A larger apical deviation relative to coronal deviation indicates angular drift of the implant body during placement, with clinical implications for proximity to vital anatomical structures.
Angular deviation	The angle (degrees, °) between the long axis of the planned implant and the long axis of the actually placed implant, determined by three‐dimensional superimposition of pre‐ and postoperative imaging. Represents rotational or tilting error relative to the intended trajectory. Clinically the most significant trueness parameter, as it directly influences prosthetic emergence profile, occlusal load distribution, and access for restorative components.
**Secondary outcomes — operative time**	
Total operative time	The elapsed time (minutes) from initiation to completion of the implant placement procedure. Definitions varied across studies: some included only active osteotomy and implant insertion, while others incorporated preoperative system calibration, patient registration, U‐shaped registration device (U‐SRD) repositioning, and setup time.
**Secondary outcomes — postoperative pain**	
Postoperative pain (VAS)	Patient‐reported pain intensity following implant surgery, measured using a 100‐mm visual analog scale (VAS) at specified postoperative time points (typically Days 0–7). A score of 0 indicates no pain; 100 indicates worst imaginable pain.
**Secondary outcomes — postoperative edema**	
Postoperative edema/swelling	Clinically assessed swelling at the surgical site following implant placement, graded on a standardized 4‐point scale: 0 = absent, 1 = mild, 2 = moderate, 3 = severe (including hematoma or ecchymosis).
**Secondary outcomes — intraoperative and postoperative complications**	
Intraoperative complications	Any unplanned adverse event occurring during the surgical procedure, including intraoperative hemorrhage, injury to adjacent teeth or neurovascular structures, implant displacement beyond the apical anatomical limit, system malfunction, or conversion to freehand surgery. Assessed as individual binary outcomes or as a composite rate.
Postoperative complications	Any adverse event occurring after surgery and prior to the follow‐up endpoint, including loss of osseointegration, early implant loss, postoperative hemorrhage, wound dehiscence, or infection.
**Secondary outcomes — early implant retention**	
Early implant retention rate	The proportion (%) of placed implants remaining in situ and clinically functional at the short‐term follow‐up time point (3–6 months), without evidence of mobility, infection, or progressive bone loss meeting failure criteria. Distinguished from long‐term implant survival, which requires follow‐up beyond 12 months.

### 2.3 Information Sources and Search Strategy

A comprehensive electronic search was conducted in major biomedical databases, including PubMed/MEDLINE, Scopus, Web of Science, and the Cochrane Central Register of Controlled Trials (CENTRAL) from database inception to March 2026, without restrictions on language.

The search strategy combined controlled vocabulary (e.g., MeSH terms) and free‐text keywords related to “robotic implant surgery,” “robot‐assisted dental implant,” “computer‐assisted implant surgery,” “dynamic navigation,” “static guidance,” and “randomized controlled trial.” The complete search strategies are presented in Supplementary Table 1. Reference lists of included studies were manually screened to identify additional eligible articles. No restrictions were placed on year of publication.

### 2.4 Study Selection

All retrieved records were imported into reference management software, and duplicates were removed. Two independent reviewers (SA and RSA) screened titles and abstracts for eligibility. Full texts of potentially relevant studies were then assessed independently against the inclusion criteria. Disagreements were resolved through discussion and consensus. The study selection process was documented using a PRISMA 2020 flow diagram.

### 2.5 Data Collection Process

Data extraction was performed independently by two reviewers (AYA and RSA) using a standardized data extraction form. Extracted data included study characteristics, sample size, participant demographics, implant site distribution, robotic system characteristics, comparator modality, follow‐up duration, and outcome measures. Trueness outcomes were extracted as mean ± standard deviation for platform, apical, and angular deviations. Secondary outcomes, including operative time, pain, edema, intraoperative and postoperative complications, and early implant retention, were also recorded. When multiple analytical approaches were reported (e.g., intention‐to‐treat and per‐protocol analyses), the primary analysis specified by the study was extracted for quantitative comparison.

### 2.6 Risk of Bias Assessment

Risk of bias was assessed independently by two reviewers (RSA and AYA) using the Cochrane Risk of Bias 2 (RoB 2) tool for RCTs (Sterne et al. 2019). The following domains were evaluated: (1) randomization process, (2) deviations from intended interventions, (3) missing outcome data, (4) measurement of the outcome, and (5) selection of the reported result. Each domain was judged as low risk, some concerns, or high risk of bias. Disagreements were resolved through discussion.

### 2.7 Data Synthesis

A qualitative synthesis was conducted for all included studies. When sufficient homogeneity in outcome reporting was present, quantitative comparisons were performed based on mean differences in positional deviations between robotic and comparator groups. Heterogeneity was assessed using the I^2^ statistic and Cochran's Q test. A fixed‐effects model was used when statistical heterogeneity was low (I^2^ < 50%), whereas a random‐effects model was applied when heterogeneity was substantial. Where pooling was not feasible, a structured qualitative narrative synthesis is provided, with explicit acknowledgment of limitations.

For Shi et al. (2025), (Shi et al. 2025) which is a three‐arm RCT (r‐CAIS, d‐CAIS, and s‐CAIS), only the two arms relevant to each pairwise comparison were included in the respective meta‐analysis. The robotic arm participants were not double‐counted across comparisons, avoiding unit‐of‐analysis errors.

### 2.8 Certainty Assessment

The certainty of evidence for outcomes was evaluated using the GRADE (Grading of Recommendations Assessment, Development and Evaluation) approach (Guyatt et al. 2008). Given that all included studies were RCTs, the certainty of evidence was initially rated as high. However, downgrading decisions were considered based on risk of bias, inconsistency, imprecision, indirectness, and publication bias.

## 3 Results

### 3.1 Search Results

**Figure 1:**
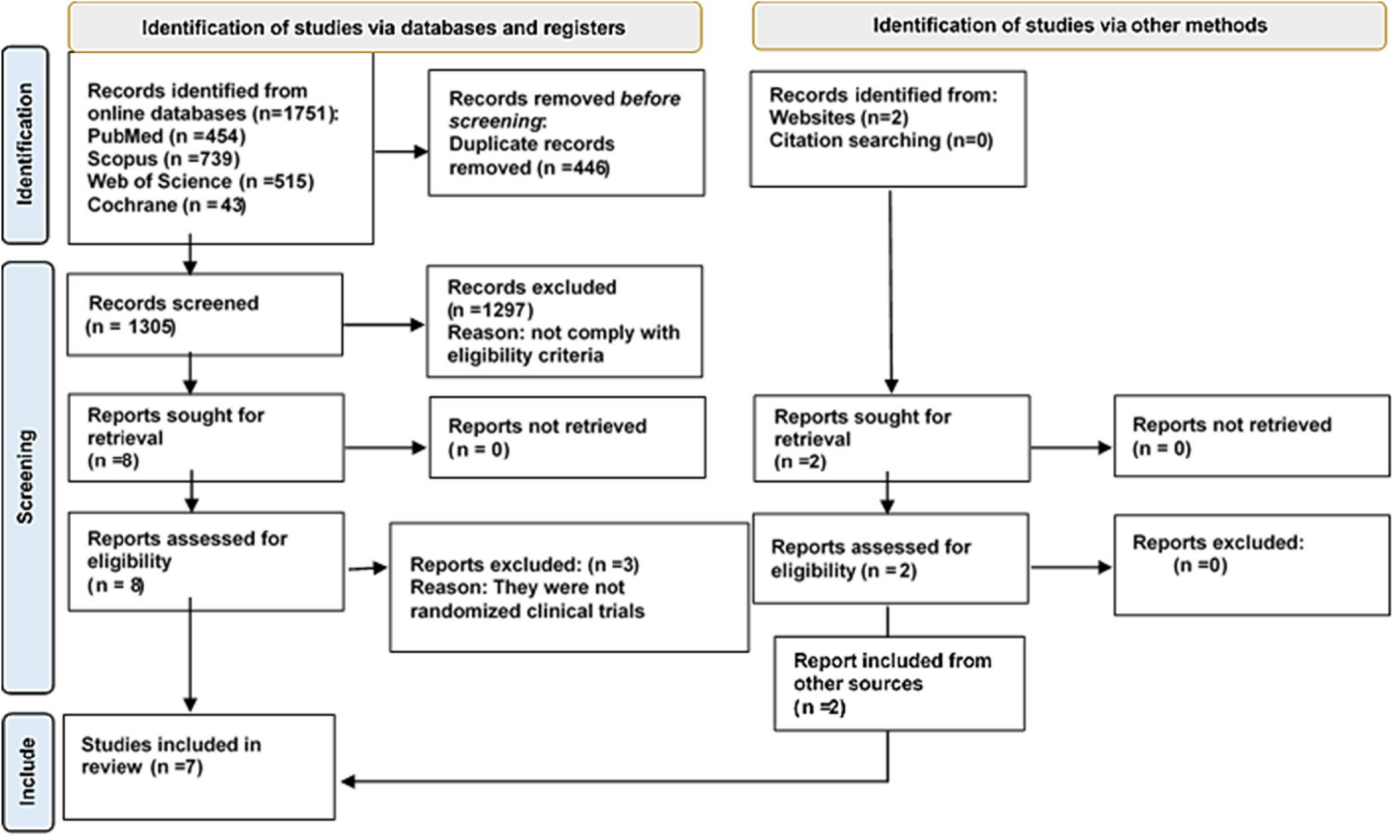
PRISMA flow chart of study selection for the systematic review and meta‐analysis.

The database search identified 1751 records, of which 446 duplicates were removed. Following title and abstract screening, 1297 records were excluded, and eight articles underwent full‐text assessment. Two additional studies were identified through manual searching. Ultimately, 7 RCTs met the inclusion criteria and were included in the qualitative synthesis, (Yang et al. 2024; Shi et al. 2024, 2025; Yang et al. 2025; Chen et al. 2025; Wei et al. 2025; Dash et al. 2026) while three studies were excluded because they were not RCTs (Wang et al. 2024; Younis et al. 2025; Zhang et al. 2024). The PRISMA flow chart presenting the screening process for this review is illustrated in Figure 1. No non‐English full‐text articles were identified meeting inclusion criteria.

### 3.2 Characteristics of the Included Studies

The seven RCTs included in this review were published between 2024 and 2026 and collectively enrolled a total of 529 implants. Six of the studies were conducted in China, (Yang et al. 2024; Shi et al. 2024, 2025; Yang et al. 2025; Chen et al. 2025; Wei et al. 2025) while one was conducted in India (Dash et al. 2026). Five trials compared r‐CAIS with FHIS, (Yang et al. 2024; Shi et al. 2024; Yang et al. 2025; Chen et al. 2025; Dash et al. 2026) one compared r‐CAIS with d‐CAIS, (Wei et al. 2025) and one was a three‐arm RCT comparing r‐CAIS with both d‐CAIS and s‐CAIS (Shi et al. 2025). Implant placement was performed in single‐tooth sites across anterior, premolar, and molar regions of both maxilla and mandible.

Six included studies reported short‐term follow‐up periods ranging from 3 to 6 months, (Yang et al. 2024; Shi et al. 2024, 2025; Yang et al. 2025; Chen et al. 2025; Wei et al. 2025) whereas one study assessed postoperative implant placement trueness obtained within 1 week and did not report a longer follow‐up period (Dash et al. 2026). The characteristics of the included studies are summarized in Table 2.

**Table 2:** Characteristics of included studies.

Study ID	Aims	Sample size	Patient age/gender	Intervention site (maxilla/mandible)/implant site	Robot user's specialty	Implant size/implant manufacturing	Groups	Bone augmentation	Loading protocol	Follow‐up (months)
Shi et al (2024)/Randomized controlled trial (RCT)/China	Compared trueness, safety, and morbidity of robotic computer‐assisted implant surgery (r‐CAIS) versus free‐hand implant surgery (FHIS)	20 implants: 20 patients/r‐CAIS: 10 patients (9 analyzed per‐protocol); FHIS: 10 patients	47 years/13 F/7 M	Maxilla: r‐CAIS 6/FHIS 5; Mandible: r‐CAIS 4/FHIS 5/Sites: anterior, premolar, and molar regions (single‐tooth sites)	Two experienced implant surgeons	Diameters: 3.3 mm, 4.1 mm; Lengths: 8 mm, 10 mm/Straumann AG, Switzerland bone and tissue level (BL/TL)	r‐CAIS vs FHIS	NR	NR	3 months (implant success evaluation)
Yang et al (2024)/RCT/China	A multicenter RCT compared the trueness of novel semi‐active r‐CAIS against conventional FHIS	140 implants: 140 patients/70 r‐CAIS; 70 FHIS	r‐CAIS 35.6 ± 12.3 yrs; FHIS 35.1 ± 12.8 yrs/43 M/97 F	Maxilla: r‐CAIS 19; FHIS 36; Mandible: r‐CAIS 51; FHIS 34/Anterior, premolar, and posterior regions (single‐tooth sites)	Experienced implant surgeons across 4 centers	Various diameters (3.3–5.0 mm) and lengths (8–12 mm)/Straumann® BL or standard plus (SP) cylindrical, non‐self‐tapping; NobelParallel Conical Connection (PCC), tapered self‐tapping	Semi‐active r‐CAIS vs FHIS	Performed when clinically indicated: r‐CAIS: 14 implants; FHIS: 20 implants	Delayed loading (prosthesis fabricated 3–6 months' post‐surgery)	3 months
Shi et al (2025) /RCT/China	Compared clinical outcomes, operator experience, and patient satisfaction across 3 CAIS modalities: r‐CAIS, dynamic computer‐assisted implant surgery (d‐CAIS), and static computer‐assisted implant surgery (s‐CAIS)	45 implants: 45 patients total (intention‐to‐treat analysis) r‐CAIS: 15 d‐CAIS: 15 s‐CAIS: 15	r‐CAIS 42.6 ± 10.9 years; d‐CAIS 42.5 ± 12.7; s‐CAIS 48.7 ± 13.6/r‐CAIS 9 M/6 F; d‐CAIS 7 M/8 F; s‐CAIS 8 M/7 F	r‐CAIS: Maxilla 6; Mandible 9 d‐CAIS: Maxilla 5; Mandible 10 s‐CAIS: Maxilla 10; Mandible 5/Premolar: r‐CAIS 1; d‐CAIS 1; s‐CAIS 2; Molar: r‐CAIS 14; d‐CAIS 14; s‐CAIS 13	Single experienced implant surgeon	Various diameters: 3.5–5.0 mm; Lengths: 8.5 mm, 10 mm, 11.5 mm/Nobel Biocare BL implants (Active)	r‐CAIS versus d‐CAIS, versus s‐CAIS	Summers' osteotome sinus floor elevation performed: r‐CAIS: 1 case d‐CAIS: 1 case s‐CAIS: 3 cases	NR	3 months (short‐term trueness and safety evaluation)
Chen et al (2025)/RCT/China	Compared the trueness, safety, and efficiency of r‐CAIS versus FHIS over a six‐month follow‐up	24 implants: 24 patients/r‐CAIS: 10; FHIS: 14	r‐CAIS: 33.2 ± 9.85; FHIS: 38.1 ± 14.8/r‐CAIS: 3 M/7 F; FHIS: 3 M/11 F	r‐CAIS: Maxilla 3; Mandible 7 FHIS: Maxilla 5; Mandible 9/anterior, premolar, and molar single‐tooth sites	Experienced implant surgeons (two operators)	Various diameters and lengths (NR)/Straumann AG, Switzerland implants (BL and TL)	r‐CAIS versus FHIS implant placement	GBR performed: r‐CAIS: 5 with/5 without FHIS: 6 with/8 without	Delayed loading	6 months
Yang et al (2025)/RCT (part of translational study)/China	Assessed the trueness and safety of a novel semi‐autonomous r‐CAIS	60 implants: 60 patients/r‐CAIS: 30 patients; FHIS: 30 patients	35.36 ± 12.05 yrs/17 M; 43 F	Maxilla and Mandible/Single‐tooth implant placement	Experienced implant surgeon with experience in the semi‐autonomous r‐CAIS surgery and more than 10 years of experience in FHIS implantation	NR/Straumann TL; Institute Straumann AG	r‐CAIS versus FHIS	NR	NR	Short‐term postoperative evaluation; 3 months (trueness and safety focus)
Wei et al (2025)/RCT/China	Compared trueness and outcomes of autonomous r‐CAIS versus d‐CAIS for standardized implant placement using the same optical tracking	40 implants: 40 patients/r‐CAIS: 20; d‐CAIS: 20	Mean age 40.8 years/20 M; 20 F	r‐CAIS: Maxilla 6; Mandible 14 d‐CAIS: Maxilla 9; Mandible 11 r‐CAIS 3 incisors, 5 premolars, 12 molars d‐CAIS 6 incisors, 6 premolars, 8 molars Single‐tooth missing	Experienced implant surgeon (trained in > 100 navigation and robotic surgeries)	Various diameters: 3.3,4.1,4.8 mm; Lengths: 8, 10, 12 mm/Straumann BL Implant System	r‐CAIS vs d‐CAIS	NR	NR	3 months (early healing and success evaluation)
Dash et al (2026)(/RCT/India	Compared trueness of semi‐active r‐CAIS versus conventional FHIS in a tertiary care Indian implantology center	200 implants: 200 patients/r‐CAIS: 100 patients; FHIS: 100 patients	18–70 years/110 M; 90 F	Max: r‐CAIS 52; FHIS 49 Mand: r‐CAIS 48; FHIS 51 Anterior: r‐CAIS 38/FHIS 36 Posterior: r‐CAIS 62/FHIS 64 (single‐tooth sites)	Single experienced implantologist	Various diameters and lengths (NR)/NR	r‐CAIS vs FHIS	NR	Delayed loading	Within 1 week (trueness); no extended follow‐up reported

Abbreviations: BL, bone level; CBCT, cone beam computed tomography; d‐CAIS, dynamic computer‐assisted implant surgery; FHIS, free‐hand implant surgery; GBR, guided bone regeneration; NR, not reported; RCT, randomized controlled trial; r‐CAIS, robotic computer‐assisted implant surgery; s‐CAIS, static computer‐assisted implant surgery.

Table 3 summarizes key characteristics and technical details of the robotic systems used in the included RCTs. The included RCTs evaluated robotic systems across three autonomy levels. Four studies used the THETA (Jianjia, China) semi‐active collaborative robot, (Yang et al. 2024; Shi et al. 2024; Yang et al. 2025; Chen et al. 2025) including one with a distinctive cantilevered all‐in‐one configuration integrating the robotic arm, camera, and software into a single unit (Chen et al. 2025). Two studies evaluated task‐autonomous systems — the Yakebot (Wei et al. 2025) and the YaZhi robot (Shi et al. 2025) — both operating with minimal intraoperative surgeon input. One study did not specify a proprietary system (Dash et al. 2026). Across all studies, optical tracking and CBCT‐based virtual planning constituted the standard technical workflow. The definition of surgeon experience varied across the included studies. One study defined experience as > 10 years of FHIS experience, (Yang et al. 2025) another as > 100 navigation and robotic procedures, (Wei et al. 2025) while the remaining studies described surgeons only as “experienced implant surgeons” without further specification (Yang et al. 2024; Shi et al. 2024, 2025; Chen et al. 2025; Dash et al. 2026).

**Table 3:** Robotic systems used in included RCTs: characteristics and technical details.

Study ID	Robot name (manufacturer)	Autonomy level	Tracking system	Registration method	Depth and angle control	Software
Shi et al (2024) /Randomized controlled trial (RCT)/China	Theta (Jianjia, Hangzhou, China)	Semi‐active: robot constrains angle; surgeon controls axial force and drill speed	Fixed infrared binocular camera (spryTrack 180, Atracsys); 850 nm; trueness 0.36 mm at < 1.4 m	U‐SRD (10 ceramic balls); 9‐point registration; threshold < 0.4 mm; verified at 6 adjacent landmarks	Arm auto‐positions at entry point; surgeon applies light axial force to drill; arm provides angular constraint and stops at planned depth	Planning: Cycad, v1, Jianjia; robotic navigation: Cypress v1, Jianjia
Yang et al (2024)/RCT/China	THETA (Jianjia, Hangzhou, China)	Semi‐active: teach button for free movement; alignment button locks arm to planned axis; surgeon guides drill axially only	Infrared binocular optical tracker; U‐tube registration with silicone impression	U‐tube registration; CBCT + STL intraoral scan imported into Cycad; 9‐point registration	Alignment button constrains axis; surgeon applies downward force; arm stops at planned depth; drill cannot deviate laterally	Planning: (Cycad, version 1, Hangzhou Jianjia Medical Technology Co. Ltd, Hangzhou, China); robotic navigation: Cypress
Shi et al (2025) /RCT/China	YaZhi (YakeRobot Technology Ltd, Beijing, China)	Task‐autonomous (Level 2): robot performs osteotomy and implant insertion; surgeon monitors only	Fixed infrared binocular camera (FusionTrack 250, Atracsys); force/torque sensor on handpiece (Mini45, ATI)	3D‐printed patient‐tracking device cemented to adjacent teeth; customized registration guide; registration guide removed after alignment	Fully autonomous drill trajectory, angle, and depth; handpiece mounted with a visual marker and force/torque sensor; surgeon monitored the autonomous procedure.	Planning: 3Shape Implant Studio; Analysis: DentalNavi 2.2 (Beijing YaKebot Technology)
Chen et al (2025)/RCT/China	THETA (all‐in‐one) (Jianjia, Hangzhou, China)	Semi‐active: arm manages angle; clinician controls vertical movement and drill power	Dual‐eye binocular camera integrated into single cantilevered unit (distinguishes from split‐machine designs)	U‐SRD (10 ceramic balls); 9‐point registration via three‐plate reflective device; verified against adjacent landmarks	Arm auto‐adjusts position and angle; clinician guides drill vertically; arm stops at predefined depth	Planning: DTX Studio Implant and Cycad v1 (Jianjia); robotic placement followed the position planned in Cycad
Yang et al (2025)/RCT (part of translational study)/China	THETA (sa‐RASS) (Jianjia, Hangzhou, China)	Semi‐autonomous: autonomous positioning and direction maintenance; drilling passively triggered by surgeon button press	Infrared binocular camera; U‐shaped silicone tube position marker; infrared reflective plate	U‐shaped silicone tube registration; CBCT + intraoral scan imported into Cypress software; physician validates registration	Autonomous angular and position control; head movement tracking; arm halts at planned depth; surgeon moves drill axially only	Planning: Cycad; Hangzhou Jianjia Medical Technology Co)
Wei et al (2025)/RCT/China	Yakebot (Beijing Yake Science and Technology Co., China)	Task‐autonomous: robot navigates autonomously along pre‐recorded extraoral trajectory; surgeon does not guide during osteotomy	Active optical tracking — same system used for both r‐CAIS and d‐CAIS groups (unique design feature eliminating inter‐system tracking error)	CBCT + intraoral scan imported into DentalNavi software; marker files exported to robotic system; surgeon pre‐records extraoral trajectory	Precise autonomous control of site, axis, and depth; robot executes osteotomy and controlled retraction independently	Planning and analysis: DentalNavi software (Beijing YaKebot Technology Co. Ltd., Beijing, China); ICP algorithm for post‐op CBCT superimposition
Dash et al (2026)/RCT/India	Unspecified semi‐active system (Neocis Yomi®; SurgiNav cited as examples; India)	Semi‐active: robotic arm provides real‐time haptic resistance, spatial boundaries, and visual alerts; surgeon performs all clinical steps	Not clearly specified; the study states that semi‐active navigation platforms compatible with Indian imaging systems were used, but the specific tracking technology was not reported	CBCT‐based virtual planning; intraoral scan integrated into navigation software; pre‐ and post‐op CBCT superimposition by blinded examiner	Mechanical constraint limits deviations from planned trajectory; haptic resistance corrects in real time; depth stop enforced by arm; visual alerts when threshold exceeded	NR

Abbreviaitons: AR, autonomous robot; CBCT, cone‐beam computed tomography; DN, dynamic navigation; FOV, field of view; Hz, hertz; ICP, iterative closest point; NR, not reported; PTRD, patient‐tracking reflective device; RS, robotic surgery; sa‐RASS, semi‐autonomous robotic‐assisted surgical system; U‐SRD, U‐shaped registration device.

Trueness evaluation methods used across the seven included RCTs are summarized in Table 4. All studies assessed implant positional trueness by superimposing preoperative plans onto postoperative imaging data. Immediate postoperative CBCT was used in five studies, (Yang et al. 2024; Shi et al. 2024; Yang et al. 2025; Chen et al. 2025; Wei et al. 2025) postoperative CBCT within 1 week was used in one study, (Dash et al. 2026) whereas one study used a delayed intraoral scan at 3 months, avoiding additional radiation exposure and metal artifacts (Shi et al. 2025). Three common deviation metrics were consistently reported across all studies: platform global deviation, apex global deviation, and angular deviation. 3D Slicer was the most frequently used superimposition and analysis platform, adopted in four of seven studies, (Yang et al. 2024; Shi et al. 2024; Yang et al. 2025; Chen et al. 2025) while two studies used proprietary software (DentalNavi) (Shi et al. 2025; Wei et al. 2025). The software used for superimposition was not specified in one study (Dash et al. 2026). Blinded outcome assessment was employed in all studies; however, interrater reliability measures were formally reported in only three studies (Shi et al. 2024, 2025; Yang et al. 2025).

**Table 4:** Methods of trueness evaluation in the included RCTs.

Study ID	Preoperative imaging	Postoperative imaging	Superimposition method	Superimposition software	Deviation metrics reported	Reference frame/coordinate system	Blinding and reliability	Assessment Timing
Shi et al (2024) /Randomized controlled trial (RCT)/China	CBCT (96 kV, 5 mA, FOV 13×13 cm, slice width 200 µm; Planmeca 3D Max ProFace)	CBCT (same exposure parameters)	3‐point superimposition in 3D Slicer; automatic selection + manual check of platform and apex points of actual implant; planned coordinates imported into pre‐surgery CBCT	3D Slicer (version 4.11)	Platform global deviation (mm); apex global deviation (mm); angular deviation (°)	Planned implant position as reference; global, horizontal, and vertical components analyzed separately	Single trained blinded investigator (ML); ICC > 0.95 for all measurements	Immediate postoperative CBCT
Yang et al (2024) /RCT/China	CBCT (96 kV, 5 mA, voxel 0.15 mm, FOV 12×9 cm)	CBCT (same exposure parameters as pre‐surgery)	3‐point alignment followed by automatic point cloud acquisition algorithm; manual adjustment if necessary; automatic recognition of actual implant position	3D Slicer software (version 4.13; Harvard, Boston, USA)	Platform global deviation (mm); apex global deviation (mm); angular deviation (°)	Platform and apex point coordinates; long‐axis comparison for angular deviation	Two blinded medical professionals from Center 1 and Center 2 (not involved in surgery); measurements performed twice; average used for analysis; passed consistency tests	Immediate postoperative CBCT
Shi et al (2025) /RCT/China	Digital intraoral scan (TRIOS 3, 3Shape) with scan body in place (SC‐S1, Beijing Segma Medical Technology); planned position from 3Shape Implant Studio	Intraoral scan at 3 months post‐surgery (TRIOS 3, 3Shape) with scan body — NOT postoperative CBCT	Alignment of postoperative intraoral scan with preoperative digital plan using DentalNavi 2.2 accuracy analysis software	DentalNavi 2.2 (YakeRobot Technology Ltd.)	Platform global deviation (mm); apex global deviation (mm); angular deviation (°)	Algebraic differential deviations reported (positive = buccal/mesial/coronal; negative = lingual/distal/apical); both global (absolute) and differential values	Independent calibrated examiner (X.‐J.F.); ICC = 0.95	3 months postoperative (intraoral scan, not CBCT — avoids metal artefacts and additional radiation)
Chen et al (2025) /RCT/China	CBCT (NewTom 5 G version FP, NewTom, Verona, Italy) + intraoral scan (TRIOS3, 3‐Shape)	CBCT (same exposure parameters; performed immediately postoperative)	Overlay of preoperative digital plan with immediate postoperative CBCT positions; ≥ 3 transformed alignment points; best‐fit algorithm alignment; alignment checked and adjusted if necessary	3D Slicer (version 4.11)	Platform global deviation (mm); apex global deviation (mm); angular deviation (°)	Platform and apex point deviations with horizontal and vertical components; long‐axis angular comparison	Trained blinded investigator (not involved in modeling); intra‐operator agreement assessed by repeat registration at 24‐h intervals	Immediate postoperative CBCT
Yang et al (2025)/RCT (part of translational study)/China	CBCT (80 kV, 6 mA, 15 s; voxel 0.15 mm; FOV 12×9 cm; grayscale 15‐bit; focal spot 0.5 mm)	CBCT (same exposure parameters)	3D Slicer fusion and calibration of planned and postoperative images; ≥ 3 alignment points; best‐fit algorithm	3D Slicer (version 4.13, Harvard)	Platform global deviation (mm); apex global deviation (mm); angular deviation (°); excessive depth deviation (implant placed deeper than planned) analyzed separately as safety indicator	Depth and lateral components analyzed separately; excessive depth defined as apically deeper than planned position	Single examiner (C.J.P.); measurements performed twice with 2‐week interval; ICC: platform 0.920, apex 0.917, angular 0.968, platform depth 0.896, platform lateral 0.761, apex depth 0.951, apex lateral 0.887	Immediate postoperative CBCT
Wei et al (2025)/RCT/China	CBCT (HiRes3D, LargeV Instrument Corp, China)	CBCT (same device, same parameters)	Iterative Closest Point (ICP) algorithm within DentalNavi software (Beijing YaKebot Technology)	DentalNavi (Beijing YaKebot Technology Co. Ltd.)	Platform global deviation (mm); apex global deviation (mm); angular deviation (°)	Center of planned implant platform = coordinate origin; X‐axis = mesial; Y‐axis = buccal; Z‐axis = coronal	Independent evaluators blinded to surgical group	Immediate postoperative CBCT
Dash et al (2026)/RCT/India	CBCT (preoperative, parameters not specified beyond CBCT‐based virtual planning)	CBCT (obtained within 1 week postoperatively)	Superimposition of pre‐ and postoperative CBCT scans (specific algorithm not detailed)	Not specified (standard CBCT analysis software implied)	Platform global deviation (mm); apex global deviation (mm); angular deviation (°)	Standard implant deviation metrics; coronal, apical, and angular components	Blinded independent examiner	Postoperative CBCT within 1 week

Operative time was reported in four studies, but the definitions varied substantially (Shi et al. 2025; Chen et al. 2025; Wei et al. 2025; Dash et al. 2026). One study measured time from local anesthesia to healing‐abutment placement, including registration, calibration, and guide checking/adaptation; (Shi et al. 2025) one recorded robotic time from patient positioning and U‐SRD repositioning to implant placement; (Chen et al. 2025) one measured active surgical time from osteotomy initiation to implant placement; (Wei et al. 2025) and one reported operative time without clearly defining the timing interval (Dash et al. 2026).

### 3.3 Risk of Bias Assessment

**Figure 2:**
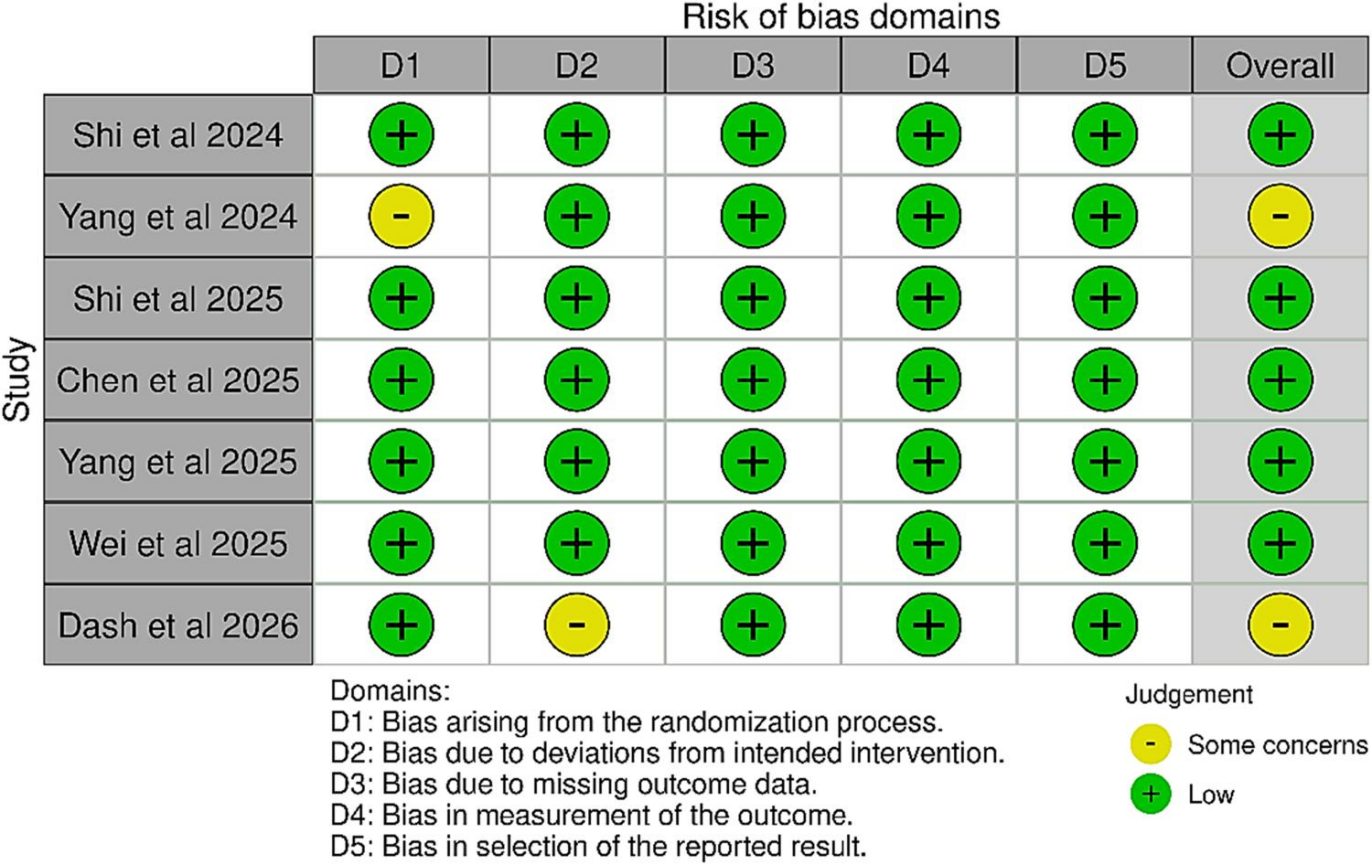
Risk of bias assessment of included RCTs according to RoB 2 tool.

Overall, the risk of bias ranged from low risk (Shi et al. 2024, 2025; Yang et al. 2025; Chen et al. 2025; Wei et al. 2025) to some concerns, (Yang et al. 2024; Dash et al. 2026) with no study judged to be at high risk of bias. The risk of bias for the included RCTs is shown in Figure 2. The main methodological limitations were minor concerns in randomization and deviations from intended interventions; however, outcomes were assessed using objective CBCT‐based measurements with minimal missing data, resulting in moderate to high internal validity.

### 3.4 Qualitative Narrative Analysis

#### 3.4.1 Primary Outcomes (Trueness)

The data reporting trueness of included RCT studies comparing r‐CAIS versus FHIS, r‐CAIS versus d‐CAIS, and r‐CAIS versus s‐CAIS are summarized in Table 5.

**Table 5:** Trueness outcomes of included studies comparing r‐CAIS versus FHIS, r‐CAIS versus d‐CAIS, and r‐CAIS versus s‐CAIS.

Study ID	Platform deviation (mean ±SD), mm	Apex deviation (mean ±SD), mm	Angular deviation (mean ±SD), degree
r‐CAIS versus FHIS			
Shi et al (2024)/Randomized controlled trial (RCT)/China	The intention‐to‐treat (ITT) analysis: Robotic computer‐assisted implant surgery (r‐CAIS): 1.27±0.46	ITT analysis: r‐CAIS 1.47±0.62	ITT analysis: r‐CAIS: 3.64°±2.99°
	Per‐protocol (PP) analysis: r‐CAIS: 1.17±0.36	PP analysis: r‐CAIS: 1.41±0.62	PP analysis: r‐CAIS: 3.46°±3.11°
	Free‐hand implant surgery (FHIS): 1.88±1.12	FHIS: 2.89±1.64	FHIS: 8.23°±7.14°
Yang et al (2024)/RCT/China	r‐CAIS: 0.76 ± 0.36 FHIS: 1.48 ± 0.93	r‐CAIS: 0.85 ± 0.48 FHIS: 2.14 ± 1.25	r‐CAIS: 2.05°±1.33° FHIS: 7.33°±4.67°
Chen et al (2025)/RCT/China	r‐CAIS: 0.70 ± 0.11 FHIS: 1.24 ± 0.59	r‐CAIS: 0.70 ± 0.12 FHIS: 2.13 ± 1.26	r‐CAIS: 1.09°±0.67° FHIS: 7.43°±6.12°
	With bone augmentation (n = 5): r‐CAIS: 0.69 ± 0.14	With bone augmentation (n = 5): r‐CAIS: 0.72 ± 0.11	With bone augmentation (n = 5): r‐CAIS: 1.32° ± 0.56°
	Without bone augmentation (n = 5): r‐CAIS: 0.72 ± 0.07	Without bone augmentation (n = 5): r‐CAIS: 0.67 ± 0.13	Without bone augmentation (n = 5): r‐CAIS: 0.86° ± 0.75°
	With bone augmentation (n = 6): FHIS: 1.07 ± 0.54	With bone augmentation (n = 6): FHIS: 1.67 ± 0.81	With bone augmentation (n = 6): FHIS: 5.59° ± 2.52°
	Without bone augmentation (n = 8):FHIS: 1.38 ± 0.63	Without bone augmentation (n = 8): FHIS: 2.48 ± 1.46	Without bone augmentation (n = 8):FHIS: 8.81° ± 7.74°
Yang et al (2025)/RCT (part of translational study)/China	r‐CAIS: 0.93 ± 0.50 FHIS: 1.45 ± 0.86	r‐CAIS: 1.07 ± 0.63 FHIS: 2.05 ± 1.16	r‐CAIS: 3.10°±1.68° FHIS: 7.94°±3.55°
Dash et al (2026)/RCT/India	r‐CAIS: 0.78 ± 0.21 FHIS: 1.42 ± 0.35	r‐CAIS: 0.91 ± 0.27 FHIS: 1.86 ± 0.44	r‐CAIS: 2.14° ± 0.62° FHIS: 4.87° ± 1.03°
r‐CAIS versus d‐CAIS			
Shi et al (2025)/randomized controlled trial (RCT)/China	r‐CAIS:1.1 ± 0.4 d‐CAIS:1.3 ± 0.6	r‐CAIS:1.5 ± 0.6 d‐CAIS:1.9 ± 0.9	r‐CAIS: 4.7°±2.5° d‐CAIS: 5.5°±3.5°
Wei et al (2025) /RCT/China	r‐CAIS: 0.70 ± 0.33 d‐CAIS: 0.77 ± 0.29	r‐CAIS: 0.76 ± 0.35 d‐CAIS: 0.80 ± 0.40	r‐CAIS: 1.01°±0.65° d‐CAIS: 1.78°±1.15°
r‐CAIS versus s‐CAIS			
Shi et al (2025) /randomized controlled trial (RCT)/China	r‐CAIS:1.1 ± 0.4 s‐CAIS:1.1 ± 0.6	r‐CAIS:1.5 ± 0.6 s‐CAIS: 2.0 ± 1.2	r‐CAIS: 4.7°±2.5° s‐CAIS: 6.2°±4.0°

### 3.5 r‐CAIS Versus FHIS

Across the five RCTs comparing r‐CAIS to FHIS, robotic guidance consistently demonstrated superior three‐dimensional trueness at all measurement levels. Platform deviations for r‐CAIS ranged from 0.70 to 1.27 mm versus 1.24 to 1.88 mm for FHIS, apex deviations from 0.70 to 1.47 mm versus 1.86 to 2.89 mm, and angular deviations from 1.09° to 3.64° versus 4.87° to 8.23°, with the direction of effect uniformly favoring r‐CAIS across the five studies and all three metrics (Yang et al. 2024; Shi et al. 2024; Yang et al. 2025; Chen et al. 2025; Dash et al. 2026). The pooled estimates and statistical significance of these differences are reported in the meta‐analysis (Section 3.5.1).

### 3.6 r‐CAIS Versus d‐CAIS

Platform deviation ranged from 0.70 to 1.10 mm in r‐CAIS and from 0.77 to 1.30 mm in d‐CAIS. Apical deviation ranged from 0.76 to 1.50 mm for r‐CAIS and from 0.80 to 1.90 mm for d‐CAIS. Angular deviation was lower in robotic arms (1.01°–4.7°) than in d‐CAIS (1.78°–5.5°) (Shi et al. 2025; Wei et al. 2025). These differences are detailed in the meta‐analysis (Section 3.5.2).

### 3.7 r‐CAIS Versus s‐CAIS

The single available r‐CAIS versus s‐CAIS comparison showed identical platform deviations between groups (1.1 ± 0.4 mm each), with marginally higher apex and angular deviations in the s‐CAIS arm alongside greater variability (Shi et al. 2025). Pooled meta‐analytic estimates for both active comparisons are presented in Section 3.5.3.

### 3.8 Secondary Outcomes

Detailed secondary and safety outcomes across included RCTs are presented in Table 6.

**Table 6:** Secondary and safety outcomes across included RCTs.

Study ID/comparison)	Operative time mean ± SD (minutes)/definition	Pain (VAS)	Edema/swelling	Intraoperative complications	Postoperative complications	Implant retention (%)	Bone/Tissue assessment	Patient‐ Reported Outcomes	Surgeon Assessment	Subgroup/Regression Analysis	Other/Unique Outcomes
Shi et al (2024) /Randomized controlled trial (RCT)/r‐CAIS vs FHIS	NR for either group	VAS (100‐mm scale) Discomfort during surgery: r‐CAIS: 1.0 (IQR 2.5); FHIS: 2.5 (IQR 4.3) Postop Days 1–7: No significant difference between groups at any time point	Not formally reported as a graded outcome; no edema adverse events noted	NR in either group: No intraoperative hemorrhage No injury to adjacent structures No nerve/tooth injury No implant displacement beyond apical limit	NR: No osseointegration failure No early implant loss No postop hemorrhage No wound dehiscence No infection	r‐CAIS: 100% FHIS: 100% (Follow‐up: not specified)	Buccal and lingual bone plate thickness assessed at 1, 3, and 5 mm apical to implant platform on post‐op CBCT. No dehiscence, perforation, or fracture of alveolar bone plate in either group	Post‐op self‐report of pain/discomfort over first week: No significant intergroup differences	NR	ITT and PP analyses reported. One case excluded from PP due to calibration error (U‐SRD dislodgement)	Conversion to freehand: not required. Safety: no issues in either group. Calibration flag: system correctly flagged calibration error in 1 robotic case
Yang et al (2024) /RCT/r‐CAIS vs FHIS	NR for either group	NR as a primary or secondary outcome	NR	NR: No implant displacement beyond apical anatomical limit No intraoperative bleeding No damage to adjacent teeth or nerves Device defect: 1 case — difficulty removing temporary adhesive resin fixing patient‐tracking device (resolved with high‐speed handpiece)	No wound dehiscence No infection No postop hemorrhage 1 implant in FHIS group failed to osseointegrate → removed at 3‐month follow‐up 0 failures in r‐CAIS group	r‐CAIS: 100% FHIS: 98.6% (1 early failure) (Follow‐up: 3 months)	NR	Not formally assessed	Surgeon satisfaction with software functionality: satisfied across all criteria. Mechanical and electrical safety confirmed. Operational stability: satisfactory throughout trial. No instrument flaws or unfavorable robotic events	Effect of implant type (self‐tapping vs non‐self‐tapping) on trueness in r‐CAIS group: Self‐tapping (Nobel PCC): significantly larger deviations vs non‐self‐tapping (Straumann BL/SP) (*p* < 0.05 for platform, apex, angular) No significant effect of: implant length, diameter, jaw location, tooth position, bone augmentation, bone density	Multicenter (4 centers); IWRS randomization. Instrument evaluation: software functionality, mechanical/electrical safety, operational stability all satisfactory
Shi et al (2025) /RCT/r‐CAIS versus d‐CAIS, versus s‐CAIS	r‐CAIS: 21.0 ± 5.5 min; d‐CAIS: 14.4 ± 2.8 min s‐CAIS: 7.4 ± 1.8 min/Time included registration and calibration	VAS (100‐mm) at 1, 2, 3, 7 days postop. No significant difference among 3 groups at any time point. Pain during surgery: r‐CAIS 0.95 (IQR 0–1.45); d‐CAIS 0.7 (IQR 0.5–1.2); s‐CAIS 0 (IQR 0–0.8); NS	NR as a separate graded outcome	2 adverse events: (1) d‐CAIS: implant placed 1.5 mm deeper than planned → manually counterrotated → stability too low → replaced with wider implant. Data included in analysis. (2) r‐CAIS: significant drill deviation after calibration → surgeon converted to freehand. Data EXCLUDED from analysis.	1 implant lost at 3 months (d‐CAIS group). 2 patients lost to follow‐up (s‐CAIS group — moved out of city). No other complications in any group	r‐CAIS: 100% d‐CAIS: 93.3% (1 implant lost) s‐CAIS: 100% (Follow‐up: 3 months)	Soft tissue microcirculation and oxygenation assessed using O2CTM device (Lea Company): Blood flow (AU), SO2 (%), rHb (AU), blood flow velocity (AU) All 3 groups: significant decrease immediately and 1 h post‐op → returned to baseline at 7 days No significant between‐group differences at any time point	OHIP‐14 (oral health‐related quality of life): s‐CAIS: significantly lower physical pain, physical disability, total score at Day 3 (*p* < 0.05) d‐CAIS: significantly higher functional limitation at Day 7 (*p* < 0.05) Willingness to receive same method again: NS between groups (r‐CAIS 11/15; d‐CAIS 13/15; s‐CAIS 11/15)	VAS assessment: d‐CAIS: significantly less difficulty reaching surgical site vs r‐CAIS and s‐CAIS (*p* = 0.004) Satisfaction with planned and actual implant position: NS among groups Difficulty of surgery: NS (*p* = 0.053) Willingness to choose same approach again: NS (r‐CAIS 8; d‐CAIS 5; s‐CAIS 7)	NR	3‐arm RCT (only study comparing all 3 CAIS modalities). Blood flow assessment unique to this study. Flapless approach. Conversion: 1 robotic case converted to freehand
Chen et al (2025) /RCT/r‐CAIS vs FHIS	r‐CAIS: 18.8 ± 4.89 min; FHIS: NR/Time counted from patient lying down to implant placement	VAS (100‐mm scale) at Days 0–7 postop. Both groups: minimal pain throughout. r‐CAIS median: 0 at all time points except Day 1 (3.0) FHIS median: 0 at all time points except Day 0 (0.5) and Day 1 (0) No significant differences between groups	Not formally graded; no edema‐related adverse events reported	NR: No intraoperative hemorrhage No injury to adjacent structures or nerves No implant displacement beyond apical limit No conversion to freehand required	NR: No loss of osseointegration No early implant loss No postop hemorrhage No wound dehiscence No infection GBR performed where bone defects present (r‐CAIS: 5; FHIS: 6); no complications	r‐CAIS: 100% FHIS: 100% (Follow‐up: 6 months)	NR	Postop VAS pain scores reported (Days 0–7). No significant intergroup differences. Patient‐reported outcomes limited to pain only	Not formally assessed. Narrative: surgeons noted shorter learning curve with robotic system as surgeon controls only vertical drilling	Trueness with vs without simultaneous GBR: r‐CAIS: no significant difference (*p* > 0.05 for all metrics) FHIS: no significant difference (*p* > 0.05 for all metrics) Robotic arm capable of locking preset angle → avoids lateral forces during narrow ridge drilling	Only study with 6‐month follow‐up. All patients underwent second‐stage surgery + crown restoration after osseointegration. Operative time definition: includes full setup (most inclusive definition among studies)
Yang et al (2025) /RCT/r‐CAIS versus FHIS	NR for either group	VAS pain scores (Days 0–7): r‐CAIS: 0 (IQR 0) at all time points FHIS: 0 (IQR 0.50) at Day 0; 0 (IQR 0.25) at Day 1; 0 thereafter No significant intergroup differences. 7 FHIS participants and 4 r‐CAIS participants reported pain Day 1; resolved within 1 day	Not formally reported as separate graded outcome. no significant events noted	NR: No intraoperative hemorrhage No injury to adjacent structures or nerves No implant displacement beyond apical limit Operational safety: surgeon expressed satisfaction; no malfunctions, stability concerns, or negative robotic incidents	1 implant in FHIS group failed to osseointegrate → removed at 3 months. 0 failures in r‐CAIS group. No wound dehiscence, infection, or postoperative hemorrhage in either group. Postoperative swelling recorded in case report form	r‐CAIS: 100% FHIS: 96.7% (1 early failure at 3 months) (Follow‐up: 3 months)	NR	Self‐reported VAS pain (Days 0–7): minimal in both groups; no significant difference. No formal patient satisfaction or OHIP assessment	Surgeon satisfaction with sa‐RASS operational safety: confirmed satisfactory. No instrument defects, work stability issues, or unfavorable events. Narrative: learning curve shorter than freehand as surgeon controls only vertical drilling	Linear regression for confounders: Group (r‐CAIS vs FHIS): significant effect on apex, lateral platform, angular deviation (*p* < 0.05) Age: significant effect on platform deviation (*p* < 0.05) and lateral platform (*p* < 0.01) Bone density: significant effect on lateral platform (*p* < 0.05) and apex depth (*p* < 0.05) Sex, implant position, diameter, length: no significant influence on apex or angular deviation	Translational study (model→animal→clinical). Excessive depth deviation analyzed separately as safety indicator: r‐CAIS: 17 cases with excessive depth (0.47 ± 0.39 mm) FHIS: 14 cases with excessive depth (0.49 ± 0.45 mm); NS difference → depth safety comparable
Wei et al (2025) /RCT/r‐CAIS vs d‐CAIS	r‐CAIS: 21.52 ± 9.81 min; d‐CAIS: 14.27 ± 3.57 min/Time measured from start of osteotomy to end of implant placement	VAS (100‐mm scale) on postop Day 1. r‐CAIS: 1 patient reported mild pain (0–50 mm range) d‐CAIS: 0 patients reported pain All others: no pain	Graded scale: 0=absent, 1=mild, 2=moderate, 3=severe Postop Day 1 and Day 7: r‐CAIS: 1 patient mild edema (grade 1) d‐CAIS: 0 patients with edema All other patients: grade 0	NR in either group: No intraoperative hemorrhage No adjacent structure injury No nerve injury No implant displacement	NR: No early implant loss or failure No infection No wound dehiscence No postop hemorrhage 1 mild edema + pain case in r‐CAIS (attributed to higher placement deviations: coronal 0.973 mm, apical 1.141 mm, angular 1.113°)	r‐CAIS: 100% d‐CAIS: 100% (Follow‐up: 3 months; all implants met ICOI success criteria) (Follow‐up: 3 months)	NR	Pain and edema only (see above). No formal patient satisfaction or OHIP questionnaire	Not formally assessed. Surgeon assessment indicated that d‐CAIS was simpler and faster to operate, whereas r‐CAIS involved additional drill‐change and data‐import steps	Linear regression analysis: trueness consistent after adjustment for covariates. No significant effect of implant diameter or length on deviation outcomes	Only study using same optical tracking system for both groups (Yakebot) — eliminates inter‐system tracking error. All implants classified as ‘success' per ICOI Pisa Consensus criteria at 3 months (pain absence, no mobility, no infection, functional restoration)
Dash et al (2026) /RCT/r‐CAIS vs FHIS	r‐CAIS: 38.5 ± 6.4 min; FHIS: 31.2 ± 5.8 min/Time includes setup time; did not clearly define the timing interval	VAS (postoperative): r‐CAIS: 2.6 ± 0.9 FHIS: 2.8 ± 1.0 *p* = 0.31 (NS) No significant difference between groups	NR as a separate graded outcome	r‐CAIS: 2 cases (2%) FHIS: 5 cases (5%) Fisher's exact test *p* = 0.24 (NS) Nature of complications not individually described	NR	r‐CAIS: 100% FHIS: 100% (Follow‐up: within 1 week for trueness; no extended clinical follow‐up reported)	NR	NR	NR	NR	Largest sample (n = 200). Least methodological detail among included studies. No long‐term follow‐up. Intraoperative complication rate numerically higher in FHIS (5%) vs r‐CAIS (2%) but NS. Only study from India (all others from China)

### 3.9 r‐CAIS Versus FHIS

#### 3.9.1 Operative Time

Operative time was inconsistently reported, and the timed interval differed across studies, limiting direct comparison. One trial reported significantly longer operative time for r‐CAIS than FHIS (38.5 ± 6.4 vs 31.2 ± 5.8 min; *p* < 0.001), but the timing interval was not clearly defined (Dash et al. 2026). Another study reported 18.8 ± 4.89 min for r‐CAIS, including setup, without an FHIS comparison (Chen et al. 2025). Three trials did not report operative time (Yang et al. 2024; Shi et al. 2024; Yang et al. 2025).

#### 3.9.2 Pain and Edema

Postoperative pain was uniformly low and comparable between groups across reporting studies. No significant intergroup differences were observed over early follow‐up, although intraoperative discomfort was numerically lower in one robotic arm (median 1.0 vs. 2.5 for FHIS) (Shi et al. 2024). Other trials similarly reported minimal pain without significant differences at any time point. Postoperative swelling was not formally graded, and no edema‐related adverse events were recorded (Yang et al. 2025; Chen et al. 2025).

#### 3.9.3 Intraoperative Complications

No serious intraoperative complications were reported in r‐CAIS versus FHIS studies. No hemorrhage, injury to adjacent structures, nerve damage, or implant displacement beyond the planned apical limit was documented. One robotic case required conversion to free‐hand placement due to a technical issue (Shi et al. 2025). A minor device‐related incident involving removal of the patient‐tracking device was resolved without consequence (Yang et al. 2024). In the largest trial, complication rates were comparable between r‐CAIS and FHIS (2% vs. 5%; *p* = 0.24) (Dash et al. 2026).

#### 3.9.4 Postoperative Complications and Implant Retention

The postoperative safety profile of r‐CAIS was consistently favorable, with no robotic implant failures reported. Two trials recorded one early osseointegration failure each in the FHIS group, yielding retention rates of 100% for r‐CAIS versus 98.6% and 96.7% for FHIS (Yang et al. 2024, 2025). All other studies reported 100% retention in both groups. No wound dehiscence, postoperative infection, or hemorrhage was documented. The longest follow‐up was 6 months, with all patients completing second‐stage surgery and crown restoration (Chen et al. 2025).

#### 3.9.5 Patient‐Reported Outcomes and Surgeon Assessment

Formal patient‐reported outcomes beyond pain VAS were not assessed in r‐CAIS versus FHIS studies. Surgeon evaluations reported satisfactory software performance, mechanical and electrical safety, and operational stability, with no instrument defects or unfavorable robotic events. Subgroup analyses suggested that self‐tapping implants were associated with greater positional deviations in the robotic group, whereas implant length, diameter, jaw location, bone density, and bone augmentation showed no significant effect (Yang et al. 2024). Regression analysis identified group allocation, patient age, and bone density as predictors of selected deviation parameters (Yang et al. 2025).

### 3.10 r‐CAIS Versus d‐CAIS

#### 3.10.1 Operative Time

Studies comparing r‐CAIS with d‐CAIS reported longer measured operative time for r‐CAIS. Mean durations were approximately 21 min for r‐CAIS and 14 min for d‐CAIS (*p* < 0.01 to *p* < 0.001) (Shi et al. 2025; Wei et al. 2025).

#### 3.10.2 Pain and Edema

No significant differences were reported over 7 days in one study, with low intraoperative VAS scores in both groups (0.95 [IQR 0–1.45] vs. 0.70 [IQR 0.5–1.2]) (Shi et al. 2025). Another study reported pain in only one r‐CAIS patient on day 1 and none in the d‐CAIS group (Wei et al. 2025). Edema was rare; only one r‐CAIS patient showed mild grade‐1 swelling, while no swelling was recorded in d‐CAIS patients (Wei et al. 2025).

#### 3.10.3 Intraoperative Complications

Intraoperative complications were uncommon in r‐CAIS versus d‐CAIS studies. One study reported no complications in either group (Wei et al. 2025). Another recorded one event per arm: depth over‐preparation in the d‐CAIS group requiring implant replacement with a wider fixture, and calibration‐related drill deviation in the r‐CAIS group requiring conversion to free‐hand placement (Shi et al. 2025).

#### 3.10.4 Postoperative Complications and Implant Retention

Postoperative outcomes were favorable in both r‐CAIS and d‐CAIS groups. One study reported 100% implant retention in both groups at 3 months, with all implants meeting ICOI success criteria (Wei et al. 2025). Another reported one implant loss in the d‐CAIS group, resulting in 100% retention for r‐CAIS versus 93.3% for d‐CAIS.

#### 3.10.5 Patient‐Reported Outcomes, Tissue Assessment, and Surgeon Assessment

Only one study included a validated patient‐reported outcome measure (OHIP‐14) and objective soft tissue assessment. Soft tissue microcirculation parameters declined immediately after surgery and at 1 h but returned to baseline by day 7, with no significant between‐group differences, indicating comparable tissue recovery across modalities (Shi et al. 2025). Another study did not assess patient‐reported outcomes but noted that surgeons considered d‐CAIS simpler and faster (Wei et al. 2025). Surgical site access was rated significantly less demanding with d‐CAIS than r‐CAIS (*p* = 0.004), although overall surgical difficulty did not differ significantly (Shi et al. 2025).

### 3.11 r‐CAIS Versus s‐CAIS

#### 3.11.1 Operative Time

Operative time differed significantly between modalities (*p* < 0.001), with r‐CAIS requiring longer operative duration than s‐CAIS (21.0 ± 5.5 vs. 7.4 ± 1.8 min) (Shi et al. 2025).

#### 3.11.2 Pain and Patient‐Reported Outcomes

Intraoperative pain was lower with s‐CAIS than r‐CAIS (median 0 [IQR 0–0.8] vs. 0.95 [IQR 0–1.45]), although postoperative pain did not differ significantly at any time point. OHIP‐14 scores favored s‐CAIS at day 3, with significantly lower physical pain, physical disability, and total scores (*p* < 0.05), suggesting a modest early quality‐of‐life benefit. Willingness to undergo the same procedure again was high and comparable in both groups (Shi et al. 2025).

#### 3.11.3 Intraoperative Complications and Safety

No intraoperative complications were reported with s‐CAIS. In the r‐CAIS group, one case required conversion to free‐hand surgery due to calibration‐related drill deviation and was excluded from per‐protocol trueness analysis. No template misfit or stabilization failure was reported with s‐CAIS (Shi et al. 2025).

#### 3.11.4 Postoperative Complications, Tissue Outcomes, and Surgeon Assessment

Implant retention at 3 months was 100% in both r‐CAIS and s‐CAIS groups, although two s‐CAIS participants were lost to follow‐up. Soft tissue microcirculation recovered to baseline by day 7, with no between‐group differences. Surgeon‐assessed implant positioning and overall difficulty were comparable between groups. Willingness to repeat the same approach was comparable (r‐CAIS: 8; s‐CAIS: 7) (Shi et al. 2025).

### 3.12 Meta‐Analysis Results

#### 3.12.1 r‐CAIS Versus FHIS

**Figure 3:**
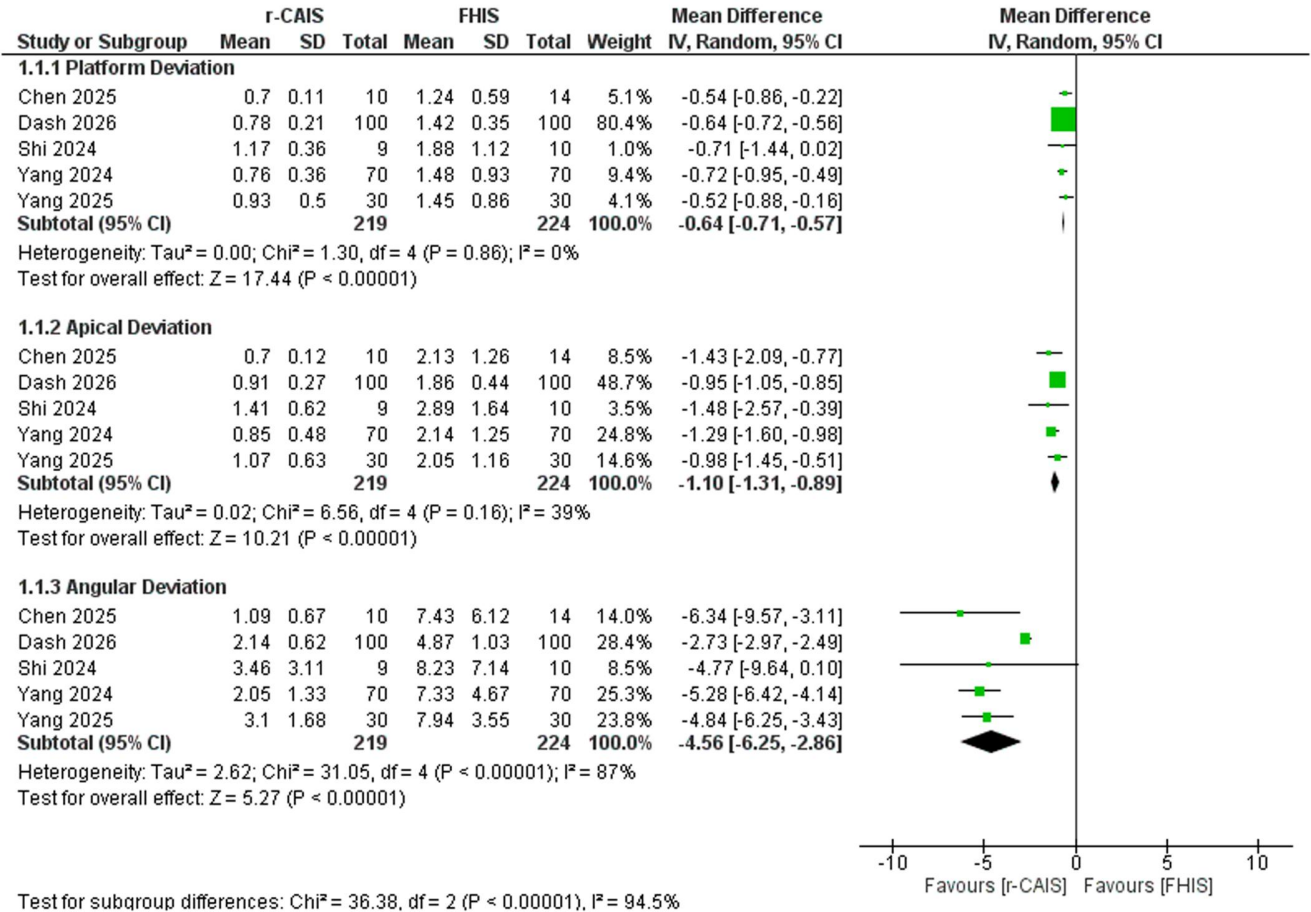
Forest plot comparing robotic computer‐assisted implant surgery (r‐CAIS) with free‐hand implant surgery (FHIS).

Meta‐analysis of trueness outcomes across five RCTs, (Yang et al. 2024; Shi et al. 2024; Yang et al. 2025; Chen et al. 2025; Dash et al. 2026) demonstrated that r‐CAIS achieved significantly greater trueness than FHIS across all evaluated parameters. For platform deviation, pooled analysis of five studies showed a statistically significant reduction in deviation favoring r‐CAIS (MD = −0.64 mm; 95% CI: −0.71 to −0.57; *p* < 0.00001), with no observed heterogeneity (I^2^ = 0%). Similarly, apical deviation was significantly lower in the r‐CAIS group compared with FHIS (MD = −1.10 mm; 95% CI: −1.31 to −0.89; *p* < 0.00001), with low‐to‐moderate heterogeneity (I^2^ = 39%). Regarding angular deviation, pooled results also favored r‐CAIS, demonstrating significantly smaller angular discrepancies than FHIS (MD = −4.56°; 95% CI: −6.25 to −2.86; *p* < 0.00001), with substantial heterogeneity detected (I^2^ = 87%). Overall, the findings indicate superior trueness of implant positioning with r‐CAIS compared with conventional freehand techniques (Figure 3).

#### 3.12.2 r‐CAIS Versus d‐CAIS

**Figure 4:**
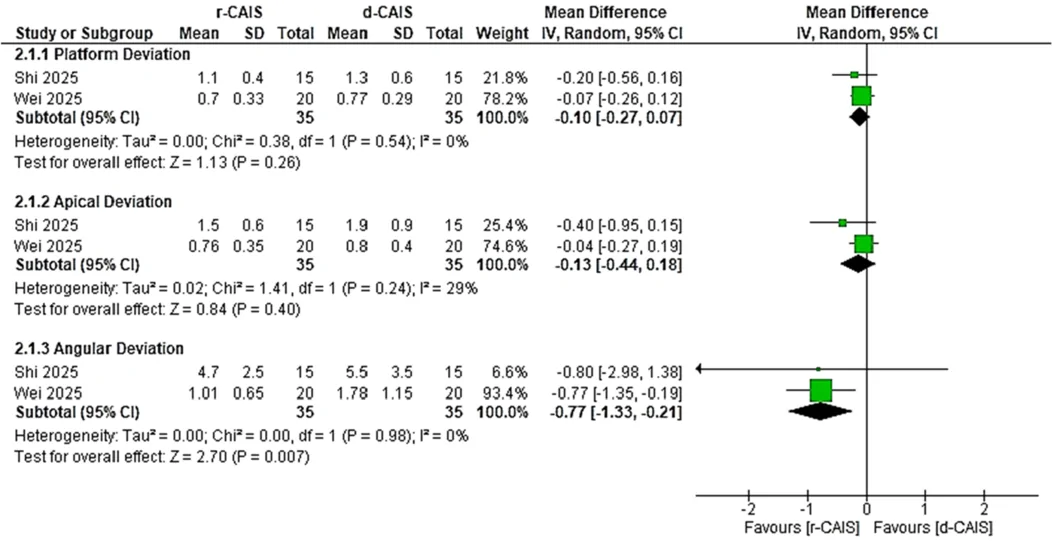
Forest plot comparing robotic computer‐assisted implant surgery (r‐CAIS) with dynamic computer‐assisted implant surgery (d‐CAIS).

Two RCTs comparing r‐CAIS with dynamic navigation were included in the meta‐analysis (Figure 4) (Shi et al. 2025; Wei et al. 2025). No statistically significant differences were observed between r‐CAIS and d‐CAIS for platform deviation (MD = −0.10 mm; 95% CI: −0.27 to 0.07; *p* = 0.26; I^2^ = 0%) or apical deviation (MD = −0.13 mm; 95% CI: −0.44 to 0.18; *p* = 0.40; I^2^ = 29%). However, angular deviation was significantly lower with r‐CAIS compared with d‐CAIS (MD = −0.77°; 95% CI: −1.33 to −0.21; *p* = 0.007; I^2^ = 0%).

#### 3.12.3 r‐CAIS Versus s‐CAIS

**Figure 5:**
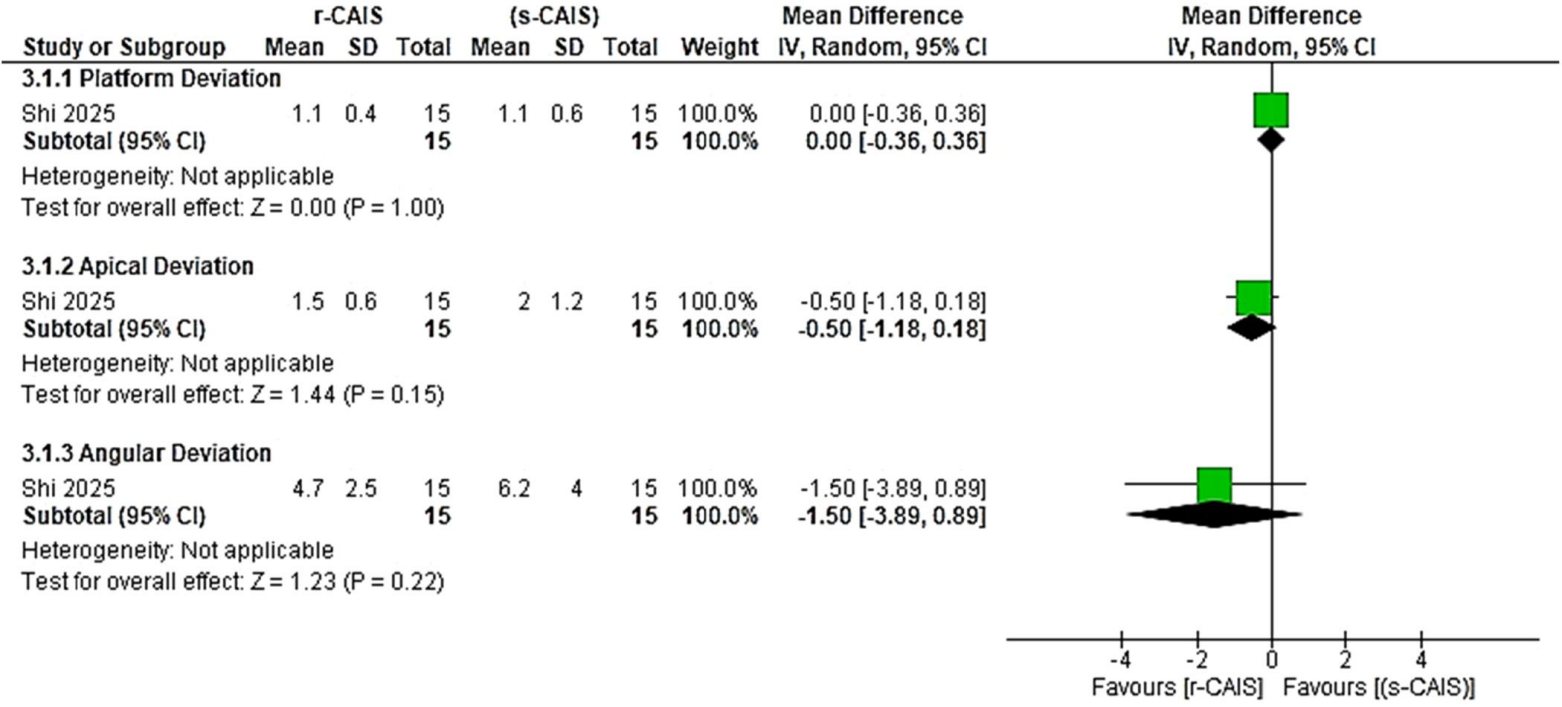
Forest plot comparing robotic computer‐assisted implant surgery (r‐CAIS) with static computer‐assisted implant surgery (s‐CAIS).

Only one RCT compared r‐CAIS with s‐CAIS (Figure 5) (Shi et al. 2025). No statistically significant differences were detected between r‐CAIS and s‐CAIS for platform deviation (MD = 0.00 mm; 95% CI: −0.36 to 0.36; *P* = 1.00), apical deviation (MD = −0.50 mm; 95% CI: −1.18 to 0.18; *p* = 0.15), or angular deviation (MD = −1.50°; 95% CI: −3.89 to 0.89; *p* = 0.22).

### 3.13 Certainty Assessment

The certainty of evidence across the three pairwise comparisons is summarized in Supplementary Table 2. For the comparison of r‐CAIS versus FHIS, the certainty of evidence was rated moderate for platform, apical, and angular deviation outcomes, reflecting some concerns regarding risk of bias in two studies, (Yang et al. 2024; Dash et al. 2026) and the geographic concentration of the evidence base, despite large and statistically significant pooled effects; heterogeneity was low for platform and apical deviations, whereas angular deviation showed substantial heterogeneity (I² = 87%). Operative time evidence was rated very low certainty due to inconsistent outcome definitions, the absence of component‐specific time reporting, and data from only four studies; early implant retention was rated low certainty owing to short follow‐up durations and a ceiling effect precluding meaningful between‐group discrimination.

For the comparison of r‐CAIS versus d‐CAIS, angular deviation was rated high certainty, supported by consistent and statistically significant pooled estimates across two low‐risk‐of‐bias trials with no heterogeneity; platform and apical deviations were rated moderate certainty, as confidence intervals crossed the null and no statistically significant differences were detected. Operative time and early retention were rated low certainty for this comparison, due to indirectness of the time metric and short follow‐up. For the comparison of r‐CAIS versus s‐CAIS, all outcomes were rated low to very low certainty, reflecting reliance on a single small trial with wide confidence intervals crossing the null for all trueness metrics. Across all comparisons, the principal drivers of downgrading were imprecision arising from small sample sizes, indirectness related to short follow‐up periods insufficient to assess long‐term implant survival, and inconsistency in operative time reporting.

## 4 Discussion

This systematic review and meta‐analysis is the first RCT‐exclusive synthesis evaluating r‐CAIS against FHIS, d‐CAIS, and s‐CAIS. r‐CAIS showed superior positional trueness versus FHIS and a significant angular advantage over d‐CAIS, whereas comparisons with s‐CAIS were non‐significant and based on limited evidence, partially rejecting the null hypothesis with respect to positional trueness.

### 4.1 Positional Trueness: r‐CAIS Versus Fhis

The r‐CAIS versus FHIS comparison is the most robustly supported finding in this review, based on 5 RCTs enrolling 444 implants (Yang et al. 2024; Shi et al. 2024; Yang et al. 2025; Chen et al. 2025; Dash et al. 2026). Meta‐analysis demonstrated significantly lower platform, apical, and angular deviations with r‐CAIS, with low heterogeneity for platform and apical deviations, indicating consistent superiority for these outcomes.

The angular deviation advantage is mechanistically well understood: robotic arms provide active angular constraint during osteotomy preparation, eliminating the cumulative directional drift inherent to free‐hand drilling. Dynamic navigation, while providing real‐time visual feedback, still relies on surgeon hand‐eye coordination and cannot eliminate angular drift from operator fatigue, limited visibility, or hand tremor (Pisla et al. 2025; Tsokkou et al. 2025; Liu et al. 2024). This distinction between constraint‐based and feedback‐based guidance explains why the angular advantage of r‐CAIS over FHIS substantially exceeds that over d‐CAIS.

Although statistically significant, no validated thresholds link angular or linear deviations to long‐term prosthetic, biological, or patient‐centered outcomes; therefore, these trueness gains cannot yet be interpreted as evidence of superior clinical effectiveness (Wang et al. 2025; Zhong et al. 2026; Sadilina et al. 2025).

Furthermore, robotic performance may vary with clinical factors. Trueness was maintained during simultaneous guided bone regeneration (GBR), (Chen et al. 2025) but greater deviations were observed with self‐tapping tapered implants than cylindrical implants, (Yang et al. 2024) and patient age and bone density significantly influenced platform and apical deviations (Yang et al. 2025). These findings suggest that robotic trueness is affected by implant design and patient characteristics.

### 4.2 Positional Trueness: r‐CAIS Versus d‐CAIS and s‐CAIS

Two RCTs compared r‐CAIS with d‐CAIS (Shi et al. 2025; Wei et al. 2025). The most robust finding was a high‐certainty reduction in angular deviation favoring r‐CAIS. Nevertheless, because this conclusion is based on only two RCTs, it should be interpreted cautiously and reassessed as further evidence emerges.

In contrast, platform and apical deviations did not differ significantly between r‐CAIS and d‐CAIS, with both outcomes rated as moderate certainty. This is mechanistically plausible because both modalities use the same CBCT‐based planning and optical registration processes, making linear trueness largely dependent on registration fidelity rather than the guidance system itself. Notably, Wei et al. (2025) used an identical optical tracking system in both groups, minimizing registration‐related confounding and providing a more direct comparison of guidance mechanisms (Wei et al. 2025).

Evidence comparing r‐CAIS and s‐CAIS is limited to a single three‐arm RCT with 15 patients per group (Shi et al. 2025). No significant differences were observed for any trueness outcome, but the evidence was of low to very low certainty and the study was not powered to demonstrate equivalence. Therefore, these findings should be interpreted as insufficient evidence rather than evidence of equivalence, highlighting the need for larger, adequately powered RCTs.

### 4.3 Operative Time

Because operative‐time definitions varied substantially across the four reporting studies (Section 3.2), (Shi et al. 2025; Chen et al. 2025; Wei et al. 2025; Dash et al. 2026) the evidence was rated as very low certainty; the available data cannot identify which workflow components account for reported differences in procedure duration or support a directional conclusion regarding operative time. Future trials should report standardized, component‐specific measures.

### 4.4 Safety, Complications, and Early Implant Retention

No included RCT reported serious intraoperative complications in robotic groups. However, six trials provided 3–6 months of follow‐up, (Yang et al. 2024; Shi et al. 2024, 2025; Yang et al. 2025; Chen et al. 2025; Wei et al. 2025) whereas one assessed trueness at 1 week without extended follow‐up (Dash et al. 2026). Therefore, postoperative findings should be interpreted as short‐term outcomes and should not be extrapolated to long‐term safety (Yang et al. 2024; Shi et al. 2024, 2025; Yang et al. 2025; Chen et al. 2025; Wei et al. 2025; Dash et al. 2026). Postoperative pain was similarly low across modalities, indicating that robotic assistance does not appear to increase early patient morbidity (Shi et al. 2024, 2025; Yang et al. 2025; Wei et al. 2025; Dash et al. 2026). The only intraoperative interruption was a conversion to free‐hand placement after a calibration‐related drill deviation, which was detected by the robotic system's built‐in safety protocol (Shi et al. 2025).

Early implant retention was high across guidance modalities and reflects early healing and osseointegration rather than long‐term implant survival or success. No implant failures were reported in robotic groups, whereas two early osseointegration failures occurred in FHIS groups (Yang et al. 2024, 2025). Nevertheless, the small number of events precludes conclusions regarding comparative biologic stability or long‐term clinical benefit.

Limited patient‐reported data suggest that workflow efficiency may influence clinical value. One trial reported better short‐term oral health–related quality of life with s‐CAIS and easier surgical access with d‐CAIS (Shi et al. 2025). These findings support broader evaluation beyond positional trueness.

### 4.5 Certainty of Evidence, Heterogeneity, and Methodological Considerations

The certainty ratings reported in Section 3.6 and Supplementary Table 2 should be interpreted in light of substantial clinical and methodological heterogeneity among the included studies.

The robotic systems differed considerably in autonomy level, arm kinematics, drill exchange workflow, and depth‐control mechanisms, with most studies evaluating the THETA semi‐active platform, (Yang et al. 2024; Shi et al. 2024; Yang et al. 2025; Chen et al. 2025) while others assessed task‐autonomous systems such as Yakebot and YaZhi, (Shi et al. 2025; Wei et al. 2025) or unspecified semi‐active platforms (Dash et al. 2026). These engineering differences likely contributed to variability in deviation outcomes beyond surgical technique alone. Methodological heterogeneity was also evident in trueness assessment protocols. Consequently, deviation values are not fully comparable across studies, and absolute numerical differences should be interpreted cautiously.

### 4.6 Clinical Implications, Strengths, Limitations, and Future Directions

The main clinical implication is that r‐CAIS improves surgical execution trueness under controlled RCT conditions; however, its financial and infrastructure requirements—including capital investment, maintenance, registration devices, and staff training—remain unquantified. Long‐term clinical outcomes, cost‐effectiveness, and learning curves require further evaluation before routine implementation can be justified.

A key strength of this review is its exclusive inclusion of RCTs, reducing bias associated with in vitro studies, non‐randomized designs, and lack of random allocation. Methodological rigor was further enhanced through PROSPERO registration, PRISMA 2020 compliance, RoB 2 assessment, prespecified analyses, and GRADE evaluation. Although no language restrictions were applied, all eligible studies were published in English; thus, any language bias likely reflects the limited availability of RCTs rather than study exclusion.

The present findings align with previous systematic reviews but provide more conservative estimates. Reviews using broader mixed‐design evidence generally reported favorable r‐CAIS trueness and, in direct comparisons with d‐CAIS, larger angular deviation advantages than the present RCT‐exclusive synthesis (Jain et al. 2023; Pisla et al. 2025; Yang and Li 2024; Khaohoen et al. 2025; Khan et al. 2024; Wang et al. 2025; Khaohoen et al. 2024; Luo et al. 2025; Wu et al. 2024; Jia et al. 2026). For example, the high‐certainty angular deviation advantage over d‐CAIS observed here (MD = −0.77°) was smaller than the −1.03° to −1.41° reported in previous mixed‐design meta‐analyses (Khan et al. 2024; Wang et al. 2025). This difference likely reflects the inflation of treatment effects under controlled laboratory conditions and non‐randomized designs.

Limitations include the small number of RCTs, modest sample sizes, short follow‐up periods, and heterogeneity in robotic systems, assessment software, implant‐related variables, trueness measurements, and operative time definitions. Implant location was not controlled in pooled analyses, despite known site‐related differences in trueness associated with bone density, access, and ridge morphology. Although two studies reported no significant effect of jaw or tooth position within r‐CAIS groups, (Yang et al. 2024; Shi et al. 2024) a meta‐regression by anatomical site was not feasible given the small number of studies. Furthermore, six of seven studies originated from a single geographic region, (Yang et al. 2024; Shi et al. 2024, 2025; Yang et al. 2025; Chen et al. 2025; Wei et al. 2025) limiting generalizability across healthcare systems, patient populations, operator training, and technology availability. Accordingly, the pooled estimates should be interpreted with caution. Finally, RCTs may inherently limit r‐CAIS evidence, as randomization requires eligibility for all comparator modalities and may exclude scenarios where robotics could be most beneficial, such as limited posterior access or immediate placement in fresh extraction sockets.

Future research should focus on multicenter RCTs with at least 12 months of follow‐up, standardized implant outcomes, and radiographic trueness assessment. Studies should incorporate the full ID‐COSM framework, including marginal bone level changes, prosthetic complications, peri‐implant tissue health, and validated patient‐reported outcomes (e.g., OHIP‐14). Comparisons between collaborative semi‐autonomous and task‐autonomous robotic systems are needed to determine the influence of autonomy level on outcomes. Standardized reporting of operative time components (registration, calibration, osteotomy, and implant insertion) and embedded cost‐effectiveness analyses should be mandatory. Learning‐curve studies are also needed to determine the number of procedures required to achieve stable robotic trueness. Consistent with recent consensus recommendations, (Sadilina et al. 2025; Yeo et al. 2025) future research should emphasize patient‐specific, indication‐driven technology selection through pragmatic, indication‐stratified trial designs. Finally, future RCTs should adopt validated operator‐experience metrics, including procedures performed, years of practice, and case complexity.

## 5 Conclusion

r‐CAIS improves implant positional trueness compared with free‐hand surgery, supported by moderate‐certainty evidence, and demonstrates a significant angular deviation advantage over dynamic navigation. Comparisons with static guidance remain underpowered, and no RCT evidence currently links trueness gains to improved prosthetic, biological, or patient‐reported outcomes. Almost all included trials followed patients for only 3 to 6 months, so the available evidence reflects early implant retention and short‐term healing rather than long‐term implant survival, marginal bone loss, peri‐implantitis risk, or prosthetic complications; evidence from trials with at least 12 months of follow‐up is still lacking and is needed before conclusions about the long‐term safety or success of r‐CAIS can be drawn. Operative time, cost‐effectiveness, and learning‐curve demands also remain insufficiently evaluated. Thus, r‐CAIS improves surgical execution trueness under controlled conditions, but long‐term multicenter RCTs with standardized clinical and patient‐centered outcomes are needed to establish its broader clinical value.

## Author Contributions

Sultan Ainoosah, Redhwan Saleh Al‐Gabri, and Ahmed Yaseen Alqutaibi made substantial contributions to the conception and design of the study. Sultan Ainoosah, Redhwan Saleh Al‐Gabri, Omar M. Alaryani, Ahmad Abdulkareem Alnazzawi, and Ahmed E. Farghal collected data. Sultan Ainoosah, Redhwan Saleh Al‐Gabri, Ahmed Yaseen Alqutaibi, and Omar M. Alaryani analyzed and interpreted data. All authors read and approved the final version of the manuscript.

## Funding

The authors have nothing to report.

## Conflicts of Interest

The authors declare no conflicts of interest.

## Systematic Review Registration

The protocol has previously been registered in the International Prospective Register of Systematic Reviews (PROSPERO) (CRD420261326729).

## Supporting information


**Table S1:** Web search keywords.

**Table S2:** GRADE evidence summary: r‐CAIS vs. FHIS, d‐CAIS, and s‐CAIS.

## Data Availability

The data that support the findings of this study are available on request from the corresponding author.

## References

[cre270460-bib-0001] Alqutaibi, A. Y. , R. S. Al‐Gabri , W. I. Ibrahim , and D. Elawady . 2025a. “Trueness of Fully Guided Versus Partially Guided Implant Placement in Edentulous Maxillary Rehabilitation: A Split‐Mouth Randomized Clinical Trial.” BMC Oral Health 25: 1680. 10.1186/s12903-025-07073-0 PMC12560370 41146170

[cre270460-bib-0002] Alqutaibi, A. Y. , H. H. Hamadallah , B. Abu Zaid , A. M. Aloufi , and R. A. Tarawah . 2025b. “Applications of Robots in Implant Dentistry: A Scoping Review.” Journal of Prosthetic Dentistry 134: 1052–1061. 10.1016/j.prosdent.2023.11.019 38087758

[cre270460-bib-0003] Chen, J. , Y. Wang , Y. Bai , et al. 2025. “Accuracy, Safety, and Efficiency in Robotic‐Assisted Vs. Freehand Dental Implant Surgery: A 6‐Month Follow‐Up Randomized Controlled Trial.” Clinical Oral Implants Research 36: 662–670. 10.1111/clr.14413 39899324

[cre270460-bib-0004] Chen, W. , K. A. Al‐Taezi , C. H. Chu , et al. 2023. “Accuracy of Dental Implant Placement With a Robotic System in Partially Edentulous Patients: A Prospective, Single‐Arm Clinical Trial.” Clinical Oral Implants Research 34: 707–718. 10.1111/clr.14083 37167364

[cre270460-bib-0005] Dash, K. C. , N. Punhani , S. Abraham , et al. 2026. “Semi‐Active Robotic Over Free‐Hand Techniques for Evaluation of Dental Implant Placement Accuracy.” Annals of African Medicine, ahead of print, April 17. 10.4103/aam.aam_149_26. 41992684

[cre270460-bib-0006] Ding, Y. , Y. Zheng , R. Chen , et al. 2023. “Accuracy of a Novel Semi‐Autonomous Robotic‐Assisted Surgery System for Single Implant Placement: A Case Series.” Journal of Dentistry 139: 104766. 10.1016/j.jdent.2023.104766 39491161

[cre270460-bib-0007] Guyatt, G. H. , A. D. Oxman , G. E. Vist , et al. 2008. “Grade: An Emerging Consensus on Rating Quality of Evidence and Strength of Recommendations.” BMJ 336, no. 6–6: 924–926. 10.1136/bmj.39489.470347.AD PMC2335261 18436948

[cre270460-bib-0008] Jain, S. , M. E. Sayed , W. I. Ibraheem , et al. 2023. “Accuracy Comparison Between Robot‐Assisted Dental Implant Placement and Static/Dynamic Computer‐Assisted Implant Surgery: A Systematic Review and Meta‐Analysis of in Vitro Studies.” Medicina 60: 11. 10.3390/medicina60010011 PMC10817552 38276045

[cre270460-bib-0009] Jia, J. , Y. Yu , X. Cao , D. Fu , C. Lv , and Y. Li . 2026. “Accuracy of Robotic Computer‐Assisted Implant Surgery in Clinical Dental Implant Placement: A Systematic Review and Meta‐Analysis.” Journal of Dentistry 166: 106321. 10.1016/j.jdent.2025.106321 41468967

[cre270460-bib-0010] Khan, M. , F. Javed , Z. Haji , and R. Ghafoor . 2024. “Comparison of the Positional Accuracy of Robotic Guided Dental Implant Placement With Static Guided and Dynamic Navigation Systems: A Systematic Review and Meta‐Analysis.” The Journal of Prosthetic Dentistry 132, no. 746: 746.e1–746.e8. 10.1016/j.prosdent.2024.02.015 38490935

[cre270460-bib-0011] Khaohoen, A. , W. Powcharoen , T. Sornsuwan , P. Chaijareenont , C. Rungsiyakull , and P. Rungsiyakull . 2024. “Accuracy of Implant Placement With Computer‐Aided Static, Dynamic, and Robot‐Assisted Surgery: A Systematic Review and Meta‐Analysis of Clinical Trials.” BMC Oral Health 24: 359. 10.1186/s12903-024-04033-y PMC10956322 38509530

[cre270460-bib-0012] Khaohoen, A. , W. Powcharoen , N. Yoda , C. Rungsiyakull , and P. Rungsiyakull . 2025. “Accuracy in Dental Implant Placement: A Systematic Review and Meta‐Analysis Comparing Computer‐Assisted (Static, Dynamic, Robotics) and Noncomputer‐Assisted (Freehand, Conventional Guide) Approaches.” Journal of Prosthetic Dentistry 134, no. 91: 91.e1‐e25. 10.1016/j.prosdent.2025.03.038 40221370

[cre270460-bib-0013] Li, J. , M. Dai , S. Wang , X. Zhang , Q. Fan , and L. Chen . 2024. “Accuracy of Immediate Anterior Implantation Using Static and Robotic Computer‐Assisted Implant Surgery: A Retrospective Study.” Journal of Dentistry 148: 105218. 10.1016/j.jdent.2024.105218 38955260

[cre270460-bib-0014] Liu, C. , Y. Liu , R. Xie , Z. Li , S. Bai , and Y. Zhao . 2024. “The Evolution of Robotics: Research and Application Progress of Dental Implant Robotic Systems.” International Journal of Oral Science 16: 28. 10.1038/s41368-024-00296-x PMC10999443 38584185

[cre270460-bib-0015] Luo, Z. , A. Li , A. Unkovskiy , et al. 2025. “Accuracy of Robotic Computer‐Assisted Implant Surgery in Clinical Studies: A Systematic Review and Meta‐Analysis.” BMC Oral Health 25: 540. 10.1186/s12903-025-05837-2 PMC11992838 40217233

[cre270460-bib-0016] Ma, W. , X. Chen , H. Liu , M. Yang , and X. Tong . 2025. “Application of Dental Implant Robots and Conventional Dental Implants in Oral Implantology: A Propensity Score Matching Study.” Srpski Arhiv za Celokupno Lekarstvo 153: 426–433.

[cre270460-bib-0017] Miadili, M. , X. Li , Y. Zhang , et al. 2025. “The Impact of Jawbone Regions (Molar Area, Premolar Area, Anterior Area) and Bone Density on the Accuracy of Robot‐Assisted Dental Implantation: A Preliminary Study.” Frontiers in Bioengineering and Biotechnology 13: 1536957. 10.3389/fbioe.2025.1536957 PMC11893848 40070547

[cre270460-bib-0018] Page, M. J. , J. E. McKenzie , P. M. Bossuyt , et al. 2021. “The Prisma 2020 Statement: An Updated Guideline for Reporting Systematic Reviews.” BMJ 372: n71. 10.1136/bmj.n71 PMC8005924 33782057

[cre270460-bib-0019] Pisla, D. , V. Bulbucan , M. Hedesiu , et al. 2025. “Accuracy of Navigation and Robot‐Assisted Systems for Dental Implant Placement: A Systematic Review.” Dentistry Journal 13: 537. 10.3390/dj13110537 PMC12651058 41294519

[cre270460-bib-0020] Qiao, S. C. , X. Y. Wu , J. Y. Shi , M. S. Tonetti , and H. C. Lai . 2023. “Accuracy and Safety of a Haptic Operated and Machine Vision Controlled Collaborative Robot for Dental Implant Placement: A Translational Study.” Clinical Oral Implants Research 34: 839–849. 10.1111/clr.14112 37309242

[cre270460-bib-0021] Sadilina, S. , K. Vietor , R. Doliveux , et al. 2025. “Beyond Accuracy: Clinical Outcomes of Computer Assisted Implant Surgery.” Clinical and Experimental Dental Research 11: e70129. 10.1002/cre2.70129 PMC12081946 40375737

[cre270460-bib-0022] Schnutenhaus, S. , M. Wagner , C. Edelmann , R. G. Luthardt , and H. Rudolph . 2021. “Factors Influencing the Accuracy of Freehand Implant Placement: A Prospective Clinical Study.” Dentistry Journal 9: 54. 10.3390/dj9050054 PMC8151810 34068734

[cre270460-bib-0023] Shi, J. Y. , B. L. Liu , X. Y. Wu , et al. 2024. “Improved Positional Accuracy of Dental Implant Placement Using a Haptic and Machine‐Vision‐Controlled Collaborative Surgery Robot: A Pilot Randomized Controlled Trial.” Journal of Clinical Periodontology 51: 24–32. 10.1111/jcpe.13893 37872750

[cre270460-bib-0024] Shi, J.‐Y. , X.‐Y. Wu , X.‐L. Lv , et al. 2025. “Comparison of Implant Precision With Robots, Navigation, or Static Guides.” Journal of Dental Research 104: 37–44. 10.1177/00220345241285566 PMC11667196 39586816

[cre270460-bib-0025] Sterne, J. A. , J. Savović , M. J. Page , et al. 2019. “Rob 2: a Revised Tool for Assessing Risk of Bias in Randomised Trials.” BMJ 366: l4898. 10.1136/bmj.l4898. 31462531

[cre270460-bib-0026] Tsokkou, S. , I. Konstantinidis , A. Keramas , et al. 2025. “Comparative Analysis of Fully Guided and Free‐Hand Orthognathic Surgery: Advancements, Precision, and Clinical Outcomes.” Dentistry Journal 13: 260. 10.3390/dj13060260 PMC12192503 40559163

[cre270460-bib-0027] Wang, J. , M. Gao , Y. Zhao , et al. 2025. “Comparison of Dental Implant Placement Accuracy Between Robotic and Static or Dynamic Computer‐Assisted Surgeries: A Systematic Review and Meta‐Analysis.” Medicina oral, patologia oral y cirugia bucal 31: e1. 10.4317/medoral.27132 PMC12743385 41108766

[cre270460-bib-0028] Wang, W. , H. Xu , D. Mei , et al. 2024. “Accuracy of the Yakebot Dental Implant Robotic System Versus Fully Guided Static Computer‐Assisted Implant Surgery Template in Edentulous Jaw Implantation: A Preliminary Clinical Study.” Clinical Implant Dentistry and Related Research 26: 309–316. 10.1111/cid.13278 37728030

[cre270460-bib-0029] Wei, H. , Z. Shu , M. Tian , Y. Ge , and D. Li . 2025. “The Accuracy of Autonomous Robotic Surgery Vs. Dynamic Navigation in Implant Placement: An Open‐Label, Double‐Arm, Randomized Controlled Clinical Trial.” Clinical Oral Implants Research 36: 1486–1497. 10.1111/clr.70024 40836378

[cre270460-bib-0030] Werny, J. G. , K. Frank , S. Fan , et al. 2025. “Freehand Vs. Computer‐Aided Implant Surgery: A Systematic Review and Meta‐Analysis—Part 1: Accuracy of Planned and Placed Implant Position.” International Journal of Implant Dentistry 11: 35. 10.1186/s40729-025-00622-w PMC12048383 40314873

[cre270460-bib-0031] Wu, X. Y. , J. Y. Shi , S. C. Qiao , M. S. Tonetti , and H. C. Lai . 2024. “Accuracy of Robotic Surgery for Dental Implant Placement: A Systematic Review and Meta‐Analysis.” Clinical Oral Implants Research 35: 598–608. 10.1111/clr.14255 38517053

[cre270460-bib-0032] Yang, F. , J. Chen , R. Cao , et al. 2024. “Comparative Analysis of Dental Implant Placement Accuracy: Semi‐Active Robotic Versus Free‐Hand Techniques: A Randomized Controlled Clinical Trial.” Clinical Implant Dentistry and Related Research 26: 1149–1161. 10.1111/cid.13375 PMC11660539 39161058

[cre270460-bib-0033] Yang, F. , J. Chen , L. Wang , and Y. Ding . 2025. “Accuracy and Safety of Implant Placement With a Novel Semi‐Autonomous Robotic‐Assisted Surgical System: A Translational Research Study.” The Journal of Prosthetic Dentistry 133: 1304–1314. 10.1016/j.prosdent.2024.10.026 39580315

[cre270460-bib-0034] Yang, J. , and H. Li . 2024. “Accuracy Assessment of Robot‐Assisted Implant Surgery in Dentistry: A Systematic Review and Meta‐Analysis.” The Journal of Prosthetic Dentistry 132, no. 747: 747.e1‐e15. 10.1016/j.prosdent.2023.12.003 38195255

[cre270460-bib-0035] Yeo, X. H. , L. J. Uei , M. Yi , et al. 2025. “Computer‐Assisted Implant Surgery: Patients' Experience and Perspectives.” Clinical and Experimental Dental Research 11: e70143. 10.1002/cre2.70143 PMC12104987 40417979

[cre270460-bib-0036] Younis, H. , B. Xu , K. Acharya , et al. 2025. “Accuracy of Robot‐Assisted Implant Surgery Versus Freehand Placement: A Retrospective Clinical Study.” International Journal of Implant Dentistry 11, no. 1: 1. 10.1186/s40729-024-00586-3 PMC11699033 39751717

[cre270460-bib-0037] Zhang, S. , Q. Cai , W. Chen , et al. 2024. “Accuracy of Implant Placement via Dynamic Navigation and Autonomous Robotic Computer‐Assisted Implant Surgery Methods: A Retrospective Study.” Clinical Oral Implants Research 35: 220–229. 10.1111/clr.14216 38033198

[cre270460-bib-0038] Zhong, F. , G. Chen , B. Sun , F. Wu , and Y. An . 2026. “Comparative Accuracy Analysis of Robotic and Static Guided Implant Surgery: A Retrospective Clinical Study.” BMC Oral Health 26: 294. 10.1186/s12903-025-07625-4 PMC12903707 41514268

