# Supplementary material for: Robotic Computer‐Assisted Dental Implant Surgery Versus Free‐Hand and Guided Techniques: A Systematic Review and Meta‐Analysis of Randomized Controlled Trials
Source: Clin Exp Dent Res. 2026 Sep 19;12(5):e70460. doi: 10.1002/cre2.70460 (PMC13589651; doi:10.1002/cre2.70460)
Supplement: Supplementary file 1 [file CRE2-12-e70460-s001.docx]

| **Supplementary Table 2**. GRADE evidence summary: r-CAIS vs. FHIS, d-CAIS, and s-CAIS | | | | | | | | | | |
| --- | --- | --- | --- | --- | --- | --- | --- | --- | --- | --- |
| **Outcome** | **Studies (n)** | **Participants** | **Study Design** | **Risk of Bias** | **Inconsistency** | **Indirectness** | **Imprecision** | **Publication Bias** | **Effect Estimate (MD, 95% CI)** | **Certainty of Evidence (GRADE)** |
| **Comparison 1: r-CAIS versus FHIS — 5 RCTs, 444 implants.**[6,13,15,16,18] | | | | | | | | | | |
| **Platform (coronal) deviation** | 5 | 444 | RCTs | Some concerns (late registration; no pre-registration)[6,18] | Not serious (I² = 0%; consistent direction across all 5 studies) | Not serious (direct human RCT evidence; single-tooth sites) | Not serious (narrow 95% CI; large pooled effect) | Undetected (all studies industry-funded or from same region; small study pool) | MD = −0.64 mm (95% CI: −0.71 to −0.57) p < 0.00001 Favors r-CAIS | **⊕⊕⊕⊝ MODERATE** |
| **Apical deviation** | 5 | 444 | RCTs | Some concerns (late registration; no pre-registration)[6,18] | Not serious (I² = 39%; directionally consistent across all 5 studies) | Not serious (direct human RCT evidence; single-tooth sites) | Not serious (CI excludes null; effect is large and consistent) | Undetected (all studies industry-funded or from same region; small study pool) | MD = −1.10 mm (95% CI: −1.31 to −0.89) p < 0.00001 Favors r-CAIS | **⊕⊕⊕⊝ MODERATE** |
| **Angular deviation** | 5 | 444 | RCTs | Some concerns (late registration; no pre-registration)[6,18] | Serious (I² = 87%; substantial between-study heterogeneity in effect magnitude, although all studies favored r-CAIS) | Not serious (direct human RCT evidence; single-tooth sites) | Not serious (95% CI excludes the null; pooled effect remains statistically significant) | Undetected (all studies industry-funded or from same region; small study pool) | MD = −4.56° (95% CI: −6.25 to −2.86); p < 0.00001; favors r-CAIS | **⊕⊕⊝⊝ LOW** |
| **Operative time** | 2 | 444 | RCTs | Some concerns (setup time included within total; no component breakdown; no pre-registration)[18] | Serious (heterogeneous definitions of 'surgical time'; different robotic systems; not pooled) | Serious (total operative time not separately reported for all studies; definitions inconsistent) | Serious (only 2 studies reported time; small total sample; wide variation in reported values) | Undetected | Not pooled — narrative only r-CAIS: 18.8–38.5 min FHIS: 31.2 min (1 study) Longer times reported with r-CAIS | **⊕⊝⊝⊝ VERY LOW** |
| **Early implant retention (3–6 months)** | 5 | 444 | RCTs | Some concerns (follow-up 3–6 months only; long-term survival not assessed) | Not serious (uniformly high retention across all studies; no heterogeneity) | Serious (short follow-up precludes conclusions on long-term implant survival or prosthetic outcomes) | Serious (rare events; ceiling effect; insufficient power to detect differences in retention) | Undetected | r-CAIS: 96.7%–100% FHIS: 98.6%–100% No significant difference between groups | **⊕⊕⊝⊝ LOW** |
| **Comparison 2: r-CAIS versus d-CAIS — 2 RCTs, 70 implants**.[14,17] | | | | | | | | | | |
| **Platform (coronal) deviation** | 2 | 70 | RCTs | Low risk (low to some concerns; blinded assessment) | Not serious (I² = 0%; consistent direction) | Not serious (direct human RCT evidence) | Serious (only 2 studies; small sample; 95% CI crosses null: −0.27 to 0.07) | Undetected (insufficient studies to assess funnel plot) | MD = −0.10 mm (95% CI: −0.27 to 0.07) p = 0.26 (NS) No significant difference | **⊕⊕⊕⊝ MODERATE** |
| **Apical deviation** | 2 | 70 | RCTs | Low risk | Not serious (I² = 29%; directionally consistent) | Not serious (direct human RCT evidence) | Serious (95% CI crosses null: −0.44 to 0.18; small sample) | Undetected | MD = −0.13 mm (95% CI: −0.44 to 0.18) p = 0.40 (NS) No significant difference | **⊕⊕⊕⊝ MODERATE** |
| **Angular deviation** | 2 | 70 | RCTs | Low risk | Not serious (I² = 0%; consistent direction and statistical significance) | Not serious (direct human RCT evidence) | Not serious (95% CI excludes null: −1.33 to −0.21; p = 0.007) | Undetected | MD = −0.77° (95% CI: −1.33 to −0.21) p = 0.007 Favors r-CAIS | **⊕⊕⊕⊕ HIGH** |
| **Operative time** | 2 | 70 | RCTs | Some concerns (setup and registration time included in total; no component-level breakdown) | Not serious (both studies show longer time with r-CAIS; consistent direction) | Serious (time includes registration/calibration overhead; not purely surgical drilling time) | Serious (only 2 studies; not pooled due to time definition heterogeneity) | Undetected | Not pooled — narrative only r-CAIS: ~21 min d-CAIS: ~14 min r-CAIS consistently longer | **⊕⊕⊝⊝ LOW** |
| **Early implant retention (3 months)** | 2 | 70 | RCTs | Low risk | Not serious (uniformly high retention) | Serious (3-month follow-up only; long-term data unavailable) | Serious (rare events; ceiling effect; underpowered to detect differences) | Undetected | r-CAIS: 93.3%–100% d-CAIS: 100% No significant difference | **⊕⊕⊝⊝ LOW** |
| **Comparison 3: r-CAIS versus s-CAIS — 1 RCT, 30 implants**.[14] | | | | | | | | | | |
| **Platform (coronal) deviation** | 1 | 30 | RCT | Low risk— blinded raters; pre-registered) | Not assessed (single study; no pooling possible) | Not serious (direct human RCT evidence; posterior premolar/molar region) | Very serious (single study; n=28; 95% CI crosses null: −0.36 to 0.36) | Undetected (cannot assess with single study) | MD = 0.00 mm (95% CI: −0.36 to 0.36) p = 1.00 (NS) No difference | **⊕⊕⊝⊝ LOW** |
| **Apical deviation** | 1 | 30 | RCT | Low risk | Not assessed (single study) | Not serious | Very serious (single study; n=28; 95% CI crosses null: −1.18 to 0.18) | Undetected | MD = −0.50 mm (95% CI: −1.18 to 0.18) p = 0.15 (NS) Trend favoring r-CAIS; NS | **⊕⊕⊝⊝ LOW** |
| **Angular deviation** | 1 | 30 | RCT | Low risk | Not assessed (single study) | Not serious | Very serious (single study; n=28; 95% CI crosses null: −3.89 to 0.89) | Undetected | MD = −1.50° (95% CI: −3.89 to 0.89) p = 0.22 (NS) Trend favoring r-CAIS; NS | **⊕⊕⊝⊝ LOW** |
| **Operative time** | 1 | 30 | RCT | Some concerns (setup/calibration time included within total duration) | Not assessed (single study) | Serious (total duration includes registration overhead; not drilling time alone) | Very serious (single study; n=28; not pooled) | Undetected | Not pooled — narrative only r-CAIS: 21.0 ± 5.5 min s-CAIS: 7.4 ± 1.8 min Significantly longer with r-CAIS (p<0.001) | **⊕⊝⊝⊝ VERY LOW** |
| **Early implant retention (3 months)** | 1 | 30 | RCT | Low risk | Not assessed (single study) | Serious (3-month follow-up only; no long-term data) | Very serious (single study; n=28; ceiling effect; rare events) | Undetected | r-CAIS: 100% s-CAIS: 100% No difference | **⊕⊝⊝⊝ VERY LOW** |

**Certainty ratings:** ⊕⊕⊕⊕ HIGH — very unlikely that further research will change confidence in the estimate; ⊕⊕⊕⊝ MODERATE — further research is likely to have an important impact; ⊕⊕⊝⊝ LOW — further research is very likely to have an important impact; ⊕⊝⊝⊝ VERY LOW — any estimate of effect is very uncertain.

**Downgrading rationale:** All evidence started at HIGH certainty (RCT design). Downgrading was applied for: (1) Risk of bias — some concerns in Yang et al. 2024 and Dash et al. 2026 (late trial registration/no preregistration); (2) Imprecision — 95% CIs crossing the null, small samples, and rare events; (3) Indirectness — short follow-up (3–6 months) precluding conclusions regarding long-term implant survival and non-standardized operative-time definitions; and (4) Inconsistency — substantial heterogeneity for r-CAIS versus FHIS angular deviation (I² = 87%) and heterogeneous operative-time reporting. Geographic concentration (6 of 7 studies from China and 1 from India) was noted as a generalizability concern but was not used as a formal GRADE downgrading criterion.

CI = confidence interval; d-CAIS = dynamic computer-assisted implant surgery; FHIS = free-hand implant surgery; GRADE = Grading of Recommendations Assessment, Development and Evaluation; MD = mean difference; NS = not significant; RCT = randomized controlled trial; r-CAIS = robotic computer-assisted implant surgery; s-CAIS = static computer-assisted implant surgery.
