# Supplementary material for: Robotic Computer‐Assisted Dental Implant Surgery Versus Free‐Hand and Guided Techniques: A Systematic Review and Meta‐Analysis of Randomized Controlled Trials
Source: Clin Exp Dent Res. 2026 Sep 19;12(5):e70460. doi: 10.1002/cre2.70460 (PMC13589651; doi:10.1002/cre2.70460)
Supplement: Supplementary file 2 [file CRE2-12-e70460-s002.docx]

**Supplementary Table 1.** Web search keywords.

| **Database** | **Full Search String** | **Results (March 2026)** |
| --- | --- | --- |
| **PubMed**  https://pubmed.ncbi.nlm. nih.gov/pubmed | (robot-assisted *[Title/Abstract] OR robotic*[Title/Abstract] OR "robot"[Title/Abstract] OR "robot guided"[Title/Abstract] OR autonomous[Title/Abstract] OR automation[Title/Abstract]) AND ("dental implant*"[Title/Abstract] OR "implant dentistry"[Title/Abstract] OR "oral implant*"[Title/Abstract] OR "implant surgery"[Title/Abstract] OR "implant placement"[Title/Abstract]) | 454 |
| **Scopus**  http://www.scopus.com | TITLE-ABS-KEY (robot-assisted* OR robotic* OR "robot" OR "robot guided" OR autonomous OR automation) AND TITLE-ABS-KEY ("dental implant*" OR "implant dentistry" OR "oral implant*" OR "implant surgery" OR "implant placement") | 739 |
| **Web of Science**  www.webofscience.com | TS=(robot-assisted* OR robotic* OR "robot" OR "robot guided" OR autonomous OR automation) AND TS=("dental implant*" OR "implant dentistry" OR "oral implant*" OR "implant surgery" OR "implant placement") | 515 |
| **Cochrane**  https://www.cochranelibrary.com | (robot-assisted* OR robotic* OR "robot" OR "robot guided" OR autonomous OR automation) AND ("dental implant*" OR "implant dentistry" OR "oral implant*" OR "implant surgery" OR "implant placement" | 43 |
